# An Ontology for Workplace Violence: Protocol to Support Cross-Sector Incident Reporting in Public Services

**DOI:** 10.2196/94392

**Published:** 2026-10-15

**Authors:** Iris Steenhout, Ronald Buyl

**Affiliations:** 1 Faculty of Life Sciences Vrije Universiteit Brussel Brussels Belgium

**Keywords:** workplace violence, public services, ontology, nomenclature, incident reporting

## Abstract

**Background:**

Underreporting is a defining problem in the documentation of workplace violence across public services. This is reinforced by inconsistent, often poor incident descriptions and the absence of a clear, shared nomenclature. Without an ontology-backed structure, similar events are recorded differently across settings or remain uncodable and effectively invisible. Moreover, workplace violence (WPV) is typically registered and studied within sector- and even organization-specific categories and local reporting logics rather than through a shared semantic framework, limiting comparability and cumulative understanding across sectors. To address this, we propose creating and publicizing a workplace violence ontology (WPV-ONTO) to unify the representation of WPV events across public services.

**Objective:**

The objective of this protocol is to describe and justify a research methodology for developing a cross-sector ontology and reference nomenclature for WPV in public services (WPV-ONTO), including the scoping review, expert consensus, and evaluation procedures used to build it. Promoting openness and high standards during its creation and encouraging its uptake once available are broader project goals that this protocol is designed to support, rather than measurable objectives that this protocol itself tests.

**Methods:**

Using Protégé and the METHONTOLOGY life-cycle framework, we will develop WPV-ONTO in Web Ontology Language. A methodologically defined, iterative workflow will combine (1) focused scoping searches in Web of Science, PubMed, APA PsycInfo, ERIC, and Sociological Abstracts; (2) structured extraction from established incident-reporting tools and code lists; and (3) expert consensus. Domain experts will use a hybrid top-down and bottom-up approach to define concepts, synonyms, and relationships based on iterative review of the scientific literature and sector-specific reporting structures. A pilot study with 6 to 8 frontline workers from 2 sectors will test the prototype reporting form against predefined usability criteria before version 1.0 release.

**Results:**

The primary output will be a comprehensive, versioned WPV-ONTO accommodating key WPV concepts relevant to public services, augmented with synonyms, definitions, and references. WPV-ONTO will include an explicit hierarchical structure and relations supporting inheritance and compositional incident encoding. Development will incorporate the perspectives of frontline workers, safety and aftercare professionals, policymakers, WPV researchers, and health and information-system experts. Expert-panel recruitment is planned to begin in March 2027, with version 1.0 expected after the 24-month development period.

**Conclusions:**

WPV-ONTO is expected to enable reasoning, inference, and consistent representation of relationships among WPV concepts for application in multiple contexts, including cross-sector reporting harmonization, software and API development, research data integration, and evaluation of prevention and aftercare initiatives. By providing a shared vocabulary and explicit structure, WPV-ONTO may also facilitate linkage with other relevant information systems, such as electronic medical records, and justice and police information systems, reducing methodological fragmentation and supporting coordinated cross-sector learning while retaining the contextual specificity necessary for public service environments.

**International Registered Report Identifier (IRRID):**

PRR1-10.2196/94392

## Introduction

### Background

Workplace violence (WPV), encompassing physical assault, verbal aggression, threats, harassment, and related hostile acts directed at workers in the course of their duties, represents one of the most widespread occupational hazards of our time. WPV exposure is common and underreported in public services such as health care, education, social welfare, law enforcement, emergency response, and public transport [1,2]. This disparity is typically ascribed to solitary or insufficiently staffed shifts, irregular working hours, and interactions with members of the public who are stressed, intoxicated, or noncompliant [3-6].

Despite its prevalence, WPV in public services remains underdocumented [7,8]. This is a direct result of how WPV is currently registered, understood, and governed across organizations and sectors. When reports are submitted, they often contain brief, inconsistent, or ambiguous descriptions. This poses limits on the reliable classification, aggregation, and comparison of these incidents [9]. Moreover, workers may refrain from reporting incidents they perceive as routine features of the job, may doubt the utility or credibility of reporting systems, or may operate within organizational cultures that tacitly normalize exposure and discourage disclosure [10,11].

The lack of a common, semantically consistent terminology for WPV worsens this underreporting problem. Similar incidents are described using different words, categorized into separate groups, and recorded through reporting methods driven by local or organizational logics, both within and across sectors [12]. An attack on a transport worker, a threatening encounter in a social welfare office, and a physical attack in an emergency room may all be recorded under completely different coding systems or not recorded at all, making cross-sector comparisons nearly impossible. This fragmentation limits the overall scientific understanding of how prevention and aftercare measures work, the role of contextual risk factors, and the mechanisms of WPV.

Significant progress has been made recently in understanding WPV in specific sectors. Studies have explored the prevalence and predictors of patient-to-staff violence in health care [13], including factors associated with WPV against health care workers in national contexts outside Belgium, such as China [14], the role of organizational and environmental factors in provoking aggressive behavior [15], and the development of sector-specific prevention programs [16,17]. Legislative frameworks, including occupational safety directives and sector-level collective agreements, have formalized the requirement to record and address WPV, but the lack of consistent terminology across sectors remains [18,19]. Knowledge is siloed, which complicates meta-analyses and system-level policy assessment because findings from one sector are rarely applicable to others due to this inconsistency.

### An Ontology for WPV

In information science, ontology provides a structured, machine-readable scheme for organizing and reasoning about a knowledge domain [20]. The ontology defines the entities, classes, and relationships that constitute the domain. In the context of WPV, ontology surpasses a basic glossary by including hierarchical structures, relationships among concepts, related contextual and environmental factors, and formal definitions grounded in evidence and expert consensus. The WPV domain is inherently multifaceted, as any incident can be characterized simultaneously by the type of act, the relationship between perpetrator and target, the physical and organizational context, the severity and impact on the worker, and the potential sector-specific circumstances surrounding the incident [21]. A shared semantic architecture is necessary to represent these aspects unambiguously, along with a shared vocabulary.

Several sector-specific classification efforts were developed in the past: the type I-IV typology by the California Division of Occupational Safety and Health [22], the Occupational Safety and Health Administration (OSHA) framework for health care settings [23], and the Violent Incident Form [24] used in UK health services. However, there is currently no publicly available, cross-sector ontology for WPV. As a result, a collection of parallel, incompatible coding schemes exists, which collectively hinder research synthesis and policy coordination.

Beyond these structural elements, the multifaceted representation of an incident also depends on characteristics of the parties directly involved. Health care settings, for example, already use risk-screening tools that record whether a patient has a known history of aggression or care needs—such as cognitive impairment or a substance-related crisis—that can precipitate aggressive behavior, information that is directly relevant to prevention and to supporting rather than solely sanctioning the person involved. Reporting behavior itself may also vary systematically with the reporting worker’s profession or experience level. Capturing such perpetrator-, target-, and reporter-level characteristics, where ethically and legally appropriate, is expected to reveal further relationships among WPV concepts that a purely act-based classification would miss; this will be considered explicitly during the conceptual boundary work described under Development of the Workplace Violence Ontology (WPV-ONTO), Step 1.

The development of the WPV-ONTO described in this protocol is envisioned as a foundational resource applicable across all sectors where WPV occurs. However, given the exceptional complexity of the public-service domain, this protocol addresses public services as the primary domain for WPV-ONTO’s initial development. We define public services here as publicly funded or publicly mandated frontline sectors in which employees interact directly with members of the public in the course of statutory duties—including health care, public transport, education, social welfare, emergency response (fire and ambulance services), and law enforcement or police—as distinct from private commercial services or public-sector office-based roles without direct public contact. The conceptual framework and methodological approach established through this work will be designed to support expansion to other sectors in later phases.

We aim to develop and share WPV-ONTO as a versioned, Web Ontology Language (OWL)–based ontology that encodes key concepts, definitions, synonyms, relationships, and hierarchical structures related to WPV in public services. WPV-ONTO has several interconnected goals: enabling consistent and comparable incident reporting across organizations and sectors; supporting the development of interoperable reporting software and APIs; facilitating integration with related information systems, such as electronic health records and justice databases; and providing a shared conceptual framework for WPV research, prevention assessment, and policy-making.

### Objectives and Anticipated Applications

The primary deliverable of this protocol is WPV-ONTO itself—the versioned, OWL-based ontology and reference nomenclature described under the Methods section. The reporting tool and application scenarios described in this subsection are anticipated downstream uses that motivate and justify the ontology’s design; developing and deploying such tools falls outside the scope of the development protocol described here and would be pursued in separate future implementation studies (see also Future Directions section).

An anticipated use of WPV-ONTO is to translate this shared conceptual structure into practical, frontline-usable tools. Simplifying incident reporting for frontline workers through digital reporting tools and mobile apps is one of the primary uses of WPV-ONTO. The time and energy required to fill out forms that are either too long, not practical for actual workplace scenarios, or lack the necessary terminology to adequately explain incidents are common reasons why workers are reluctant to report WPV [11,12]. An ontology-enabled reporting app can tackle these issues. Ontology-enabled incident reporting has already been shown to be technically feasible in an adjacent domain: Fan et al [25] developed a Hospital Incident Reporting Ontology to support cross-system comparison of patient-safety incident reports; to our knowledge, no equivalent tool has yet been built specifically for cross-sector WPV reporting, and the application envisaged here would extend this general approach to that purpose. Users should be able to effortlessly convert their experiences into structured, comparable data without technical skills, by describing situations in simple, context-aware language. Controlled vocabularies from WPV-ONTO enable smart form filling, allow filtering incident types by sector and role, and support instant report classification at the point of entry. Apps built on ontologies can aid follow-up care by escalating incidents when their severity reaches certain thresholds or automatically connecting incident reports to appropriate assistance options. This way, WPV-ONTO will be the center of an active, worker-centered system for reporting, response, and recovery. Integrating it into specially designed reporting tools is one of the most immediately effective ways to promote practical adoption.

## Methods

The process for creating a comprehensive domain-specific ontology of WPV-ONTO is described in detail below.

### Development of the WPV-ONTO

#### Overview

The development of WPV-ONTO will use a hybrid top-down and bottom-up approach, starting with the most abstract, cross-sector concepts essential for representing WPV in public services and then refining them into more specific subconcepts. This study will use the results from our extensive literature review, including the scoping review findings presented later in this paper, and will be supplemented by a targeted review of recognized classification and terminology resources that provide important references for WPV-related content (including *International Classification of Diseases, 11th Revision* [ICD-11] [26] and Systematized Nomenclature of Medicine—Clinical Terms [SNOMED CT] [27]). During its development, WPV-ONTO will be guided by an interdisciplinary expert panel (see Stakeholder Governance: Iterative Concept Refinement and Consensus Resolution section). The ontology will be created in OWL [28] using the Protégé editor [29], enabling clear hierarchies and formally defined relationships that can be immediately used for consistent reporting, interoperable APIs, and comprehensive cross-sector analysis. This workflow follows the general ontology development life-cycle described by METHONTOLOGY [30]: steps 1 and 2 correspond to its specification and conceptualization activities, steps 3 and 4 to iterative conceptualization and formalization, and the Evaluation and Maintenance and Further Development sections described later in this section correspond to its verification or validation and maintenance activities, respectively.

The 4 steps in building the WPV-ONTO are outlined below (Figure 1).

**Figure 1:**
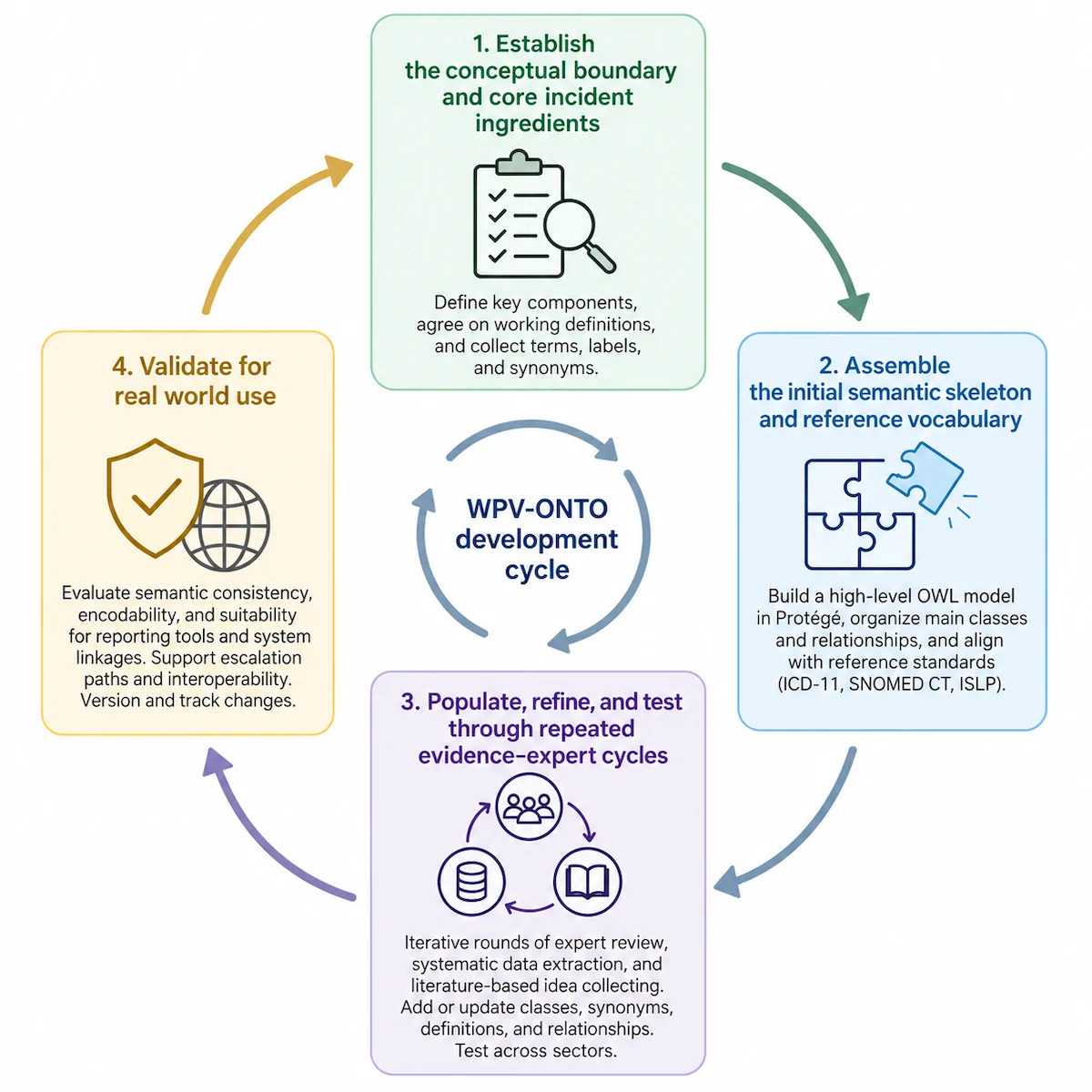
Iterative workflow for developing WPV-ONTO. The process moves from (1) defining the scope and core incident ingredients to (2) constructing an initial OWL framework, followed by (3) successive cycles using literature, reporting tools or code lists, and expert consensus, and (4) validation for real-world use. ICD-11: International Classification of Diseases, 11th Revision; ISLP: Integrated System for the Local Police; OWL: Web Ontology Language; SNOMED CT: Systematized Nomenclature of Medicine—Clinical Terms; WPV-ONTO: workplace violence ontology.

#### Step 1: Establish the Conceptual Boundary and Core Incident Ingredients

Before determining what defines WPV in a public-service setting, the expert group will catalog the key components required for sector-agnostic event description. This includes outlining key incident aspects (eg, type of aggression, roles of involved parties, setting and context, consequences or impact, and response or follow-up)—including, where ethically and legally appropriate, perpetrator- and target-related characteristics such as documented history of aggression or relevant care needs, and reporter-related characteristics such as profession, role, or experience level that may influence reporting behavior—agreeing on working definitions, and gathering potential labels and synonyms based on the scoping searches and the team’s sectoral expertise.

#### Step 2: Assemble the Initial Semantic Skeleton and Reference Vocabulary

To facilitate inheritance and incident composition, we will first build a high-level OWL model in Protégé, organizing the domain around a few main classes and relationships. External standards, such as ICD-11, SNOMED CT, and the Integrated System for the Local Police (ISLP), the reporting and operations system used by the Belgian police, will be used to improve WPV interoperability, and terms will be aligned with them. However, attention will be focused on cases where the public-service WPV domain needs concepts or levels of detail that are difficult to represent in those systems.

#### Step 3: Populate, Refine, and Test Through Repeated Evidence-Expert Cycles

Through iterative rounds of (1) expert consensus review, (2) systematic extraction from industry-standard reporting systems and code lists, and (3) targeted literature-based concept collecting (Web of Science/PubMed/APA PsycInfo/ERIC/Sociological Abstracts), WPV-ONTO will be developed. Each round, classes, synonyms, definitions, and relationships will be added or updated. It will be verified whether the model can accurately encode real-world event descriptions across different sectors.

#### Step 4: Validate the Ontology for Real-World Use

The developing WPV-ONTO will be evaluated for semantic consistency (such as hierarchies and relationships), encodability (can frontline scenarios be represented without resorting to “miscellaneous” categories?), and suitability for reporting tools (including severity-based escalation paths and potential linkage with related systems like health records or justice or police data structures). We will keep track of changes through versioning.

### Stakeholder Governance: Iterative Concept Refinement and Consensus Resolution

An expert panel of senior practitioners and researchers will collaborate to define the scope of WPV-ONTO and interpret evidence from the project’s targeted scoping searches. The team will draw on expertise in public-service practice, occupational health and safety, legal and policy fields, and informatics to ensure that the ontology is both practically credible and technically interoperable across different environments. Participants will be recruited purposefully through the research team’s established networks, including the national committee on aggression against public-service workers outlined in the Results section, pertinent professional associations, and direct invitations to acknowledged experts in these fields. Recruitment will aim for balanced representation across sectors and disciplines rather than a fixed quota. Before participating, each participant will receive a participant information sheet (Multimedia Appendix 1) and provide informed consent using a dedicated form (Multimedia Appendix 2). Anonymized participant characteristics (eg, sector and years of relevant experience) will be collected and reported in aggregate, for example, as a summary table in the resulting publication, to allow readers to assess the group’s composition and credibility.

As an illustrative target composition, the panel will aim to include a minimum of 30 participants, with at least 2 participants from each of the following sectors or disciplines: health care, public transport, education, social welfare, law enforcement, occupational health and safety, legal or policy, and health and information informatics, to ensure that no single sector or disciplinary perspective dominates concept development. Where feasible, selection will also aim for a mix of frontline and management-level experience and a reasonable gender balance as additional means of limiting selection bias. These target figures are illustrative and will be finalized and reported against the actual recruited panel in the resulting publication.

The team will meet regularly, preferably every 6-8 weeks, over 24 months (Figure 2), to refine conceptual categories, definitions, and relationships. Each session will review the current evidence, analyze newly identified concepts from reporting tools and code lists, and assess whether these concepts can be applied consistently without overlooking key contextual details.

**Figure 2:**
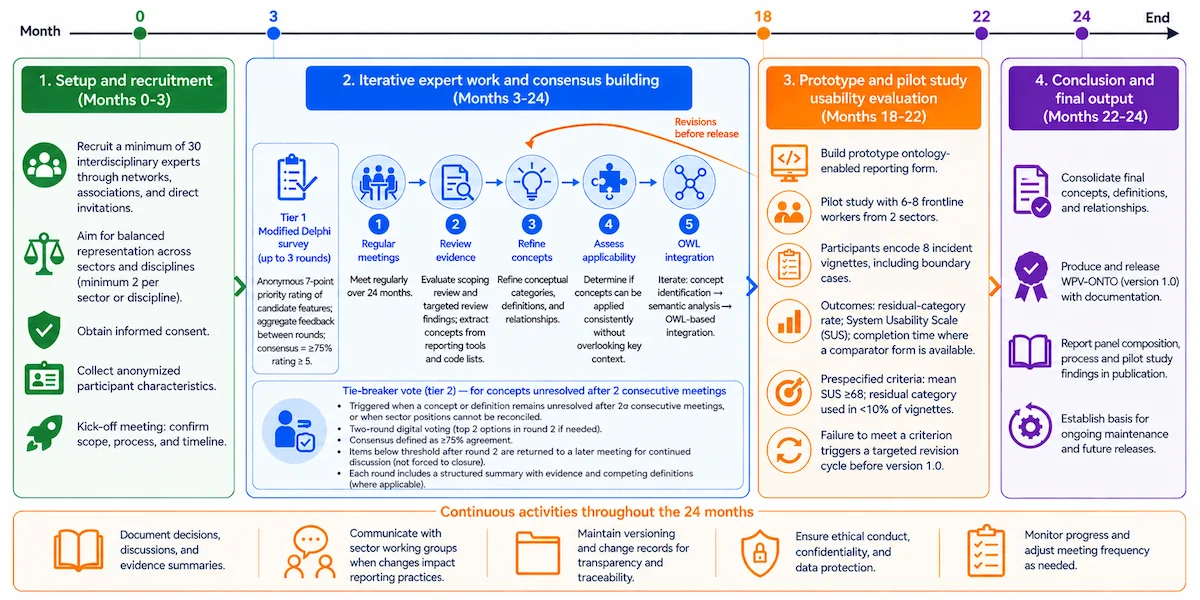
Overview of study timelines. OWL: Web Ontology Language; WPV-ONTO: workplace violence ontology.

Consensus will be built through a 2-tier, modified Delphi procedure, reported in accordance with CREDES (Guidance on Conducting and Reporting Delphi Studies) [31]. In the first tier, all panel members complete a multiround Delphi survey (up to 3 rounds; the round 1 instrument is provided in Multimedia Appendix 3), in which candidate features of WPV-ONTO—its definitional scope, structural design, and cross-sector requirements—are rated individually and anonymously on a 7-point priority scale, with optional free-text justification. The first round entails 21 items that are distributed across the themes in Table 1.

Consensus is defined a priori as at least 75% of respondents rating an item 5 or higher (moderate to essential priority). After each round, participants receive a structured, anonymized summary of aggregate ratings and comments, together with a brief evidence summary drawn from the scoping review and reporting-tool extraction and, where applicable, competing definitions identified across sources; items not reaching consensus are rerated in the subsequent round, alongside new items proposed by panelists. The procedure departs from the classical Delphi in that panel members are invited to face-to-face meetings: the survey rounds establish priorities, which the recurring stakeholder governance meetings then operationalize during ontology development. In the second tier, if a specific concept or definition remains unresolved after 2 consecutive meetings, or when sector representatives hold positions that discussion alone cannot reconcile, it is settled by a 2-round digital vote (an initial round across all options and, if needed, a second round limited to the 2 most supported), applying the same 75% threshold; items still below threshold are flagged for continued discussion at a later meeting, or deferred to a subsequent ontology release, rather than forced to closure. Evidence from scoping searches will help identify candidate concepts and recurring ambiguities across key WPV subdomains, across the key WPV subdomains identified during data charting. To ensure that WPV-ONTO continues to serve as a consistent, versioned resource for incident reporting across sectors and interoperable tools, the expert group will thoroughly evaluate these candidates before adding them to the ontology through iterations of concept identification, semantic analysis, and OWL-based integration. At the end of the project, WPV-ONTO (version 1.0) will be released together with accompanying documentation.

**Table 1:** Content of the Delphi round 1 instrument (Multimedia Appendix 3): themes and core measurement items.

Theme	Questions	Content
Theme 1	Q01-Q07	Conceptual and definitional scope: What WPV-ONTO should define, include, and exclude
Theme 2	Q08-Q14	Ontological structure and relationships: how WPV-ONTO^a^ should be structured, encoded, and governed
Theme 3	Q15-Q21	Cross-sector applicability and reporting practice: fit with sector needs, information systems, law, and governance

^a^WPV-ONTO: workplace violence ontology.

### Scoping Review

We will perform a scoping review of peer-reviewed literature as part of the iterative evidence-expert cycles described earlier. This approach closely follows the method used by Golec et al [32] and supports evidence-based concept development for WPV-ONTO. Following the steps in the methodological framework proposed by Arksey and O’Malley [33], and updated by Levac et al [34], we will (1) develop research questions based on the PICOS (population, intervention or exposure, comparator, outcome, study design) framework [35]. Specifically, the guiding research question is: “How is workplace violence conceptualized and classified in the peer-reviewed literature on public-service sectors, and what terminology and classification structures have been proposed or used?” The corresponding PICOS elements are as follows: population—workers, or WPV incidents involving workers, in the public-service sectors defined earlier; intervention or exposure—not applicable in the traditional sense, as this is a conceptual or classification review, so the feature of interest is the presence of a WPV definition, taxonomy, coding scheme, or nomenclature; comparator—not applicable, or, where present, comparison between existing classification systems; outcome—reported concepts, definitions, synonyms, classification structures, and boundary ambiguities relevant to WPV; and study design—any peer-reviewed study design (quantitative, qualitative, mixed methods, or conceptual or methodological) that proposes, describes, evaluates, or applies a WPV definition, taxonomy, or classification, consistent with the broad, concept-focused scope typical of scoping reviews. We will (2) search the Web of Science, PubMed, APA PsycInfo, Sociological Abstracts, and ERIC databases for relevant studies; (3) screen studies according to inclusion and exclusion criteria; (4) chart data by reviewing relevant papers; and (5) collect and analyze the results.

Consistent with the Arksey and O’Malley or Levac framework’s descriptive, non–meta-analytic orientation, the analysis will combine a numerical summary (eg, counts of concepts and terms by sector) with a qualitative content analysis of charted data. Extracted definitions and boundary cases will be grouped into recurring conceptual clusters through iterative coding by 2 reviewers (IS and RB). This qualitative analysis, rather than a formal thematic-analysis framework, will directly inform the candidate concepts and ambiguities fed into the expert-consensus process described under the Stakeholder Governance: Iterative Concept Refinement and Consensus Resolution section. As this scoping review is one component of a broader, multimethod ontology-development protocol, it will be reported according to the PRISMA-ScR (Preferred Reporting Items for Systematic Reviews and Meta-Analyses Extension for Scoping Reviews) framework [36]; the reporting standards for the other protocol components (ontology development, expert consensus, and usability evaluation) are described separately under the Reporting Standards section. The scoping review protocol, including the finalized search strings and eligibility criteria, will be registered prospectively on the Open Science Framework before the database searches are run.

The goal of these searches is to better understand the meaning, operationalization, categorization, and documentation of WPV across various public service agencies. When combined, they will reveal potential ideas, labels, synonyms, and ongoing uncertainties that need clarification before consistent cross-sector registration can be achieved. The search strategy will incorporate WPV umbrella terms (eg, workplace violence, workplace aggression, occupational violence, and third-party violence) along with terms related to classification and registration (eg, taxonomy, typology, classification, nomenclature, definition, framework, reporting, incident report, coding, and instrument). Sector terms pertaining to public services (eg, healthcare, emergency services, police, fire services, education, social welfare, and public transport) will be used to ensure relevance. As an illustrative, replicable example, the draft PubMed search string is: (“workplace violence”[tiab] OR “workplace aggression”[tiab] OR “occupational violence”[tiab] OR “third-party violence”[tiab]) AND (taxonomy[tiab] OR taxonomies[tiab] OR typology[tiab] OR typologies[tiab] OR classification[tiab] OR classifications[tiab] OR nomenclature[tiab] OR definition[tiab] OR framework[tiab] OR “incident report”[tiab] OR “incident reports”[tiab] OR “incident reporting”[tiab] OR coding[tiab] OR instrument[tiab]) AND (healthcare[tiab] OR “emergency services”[tiab] OR police[tiab] OR “fire service”[tiab] OR “fire services”[tiab] OR education[tiab] OR “social welfare”[tiab] OR “public transport”[tiab] OR “public transportation”[tiab]), limited to 2020-present. No single database provides comprehensive coverage of this cross-sector domain. The search will include APA PsycInfo for psychological literature on aggression, incivility, and harassment related to the conceptual boundary work. ERIC will be searched for education sector representation, less covered in biomedical databases. Sociological Abstracts will cover social science and social work literature. Occupational safety and health will be addressed through a targeted review of standards, guidance, and gray literature, as principal sources are institutional guidance and standards rather than scientific literature. The draft search strings for these databases and Web of Science Core Collection are in Multimedia Appendices 4 and 5.

This draft will be piloted and refined for precision or recall before the final search is run. Our focus will be on full-text, peer-reviewed English-language research published since 2020, covering empirical studies using quantitative or qualitative methods, as well as conceptual or methodological papers that propose or discuss WPV definitions or classifications; studies that report only clinical outcomes without addressing definition or classification will be excluded. Relevance to public services will be determined by whether the study population or setting falls within the public-service sectors defined earlier. This reflects recent advancements and provides adequate evidence. The 2020 cutoff applies to this systematic database search only; it is not intended to exclude seminal older classification and typology papers (eg, the type I-IV typology and OSHA framework already discussed in the Introduction section), which are captured instead through the separate, nondatabase-restricted targeted review of recognized classification and terminology resources described in the Development of the WPV-ONTO section. Exclusions include non-peer–reviewed publications, such as editorials, letters to the editor, comments, and press stories. Two reviewers will independently assess each paper, and any discrepancies will be resolved through discussion.

Throughout the data charting process, we will systematically extract the WPV terms used for each source, delineate the definitional boundaries provided, and identify any structured categorization of incidents. This encompasses labels and synonyms for various forms of aggression, classifications of actor and target roles, contextual descriptors including setting and task, specified means or modality, impact domains such as physical, psychological, and operational, as well as elements related to organizational response or aftercare. Particular focus will be directed toward the recurring ambiguities surrounding boundaries (eg, threat vs intimidation), as these represent frequent areas where incident reporting may exhibit inconsistencies. It is essential for WPV-ONTO to establish clear distinctions or to implement principled modeling approaches in these contexts.

Records retrieved from all 5 databases will be exported to a reference manager, automatically deduplicated, and manually checked for residual duplicates. Counts before and after deduplication, and per database, will be reported in the PRISMA-ScR flow diagram accompanying the scoping review.

### Targeted Review of Standards, Guidance, and Gray Literature

Peer-reviewed databases do not index the organizational literature in which WPV is defined and recorded. Authoritative sources in this area include standards, guidelines, and specifications issued by competent authorities and sector-specific bodies. The fact that this is currently done in such a fragmented way, with each domain operating within its own knowledge silo, forms the basis for this protocol. We will therefore conduct a targeted review selected according to the following criteria:

- The literature is published by an international standards body, a European Union agency, an intergovernmental organization, a national competent authority, or a recognized sector federation.
- The publication focuses on WPV and psychosocial risks within the context of public service, or on a definition or reporting framework applicable to it.
- The data are publicly available.

Belgian sources will be searched in Dutch, French, German, and English.

The institutional literature is highly fragmented. The core set, as described in Table S1 in Multimedia Appendix 5, will serve as the starting point. Additional sources may be identified during the process through references in existing documents or through suggestions from members of the expert panel. Each source identified in this way will be assessed against the same inclusion criteria (see Scoping Review section) and recorded in a log (Table S2 in Multimedia Appendix 5) containing the date of addition, the method through which it was identified, and the outcome of the criteria assessment. This log, including both the core set and any additional sources, will be published as an appendix to the final review. In this way, every source included in the final set will be traceable. The sources will be screened using fixed search terms (workplace violence, aggression, harassment, third-party violence, psychosocial risk, and incident reporting). For each source, 50 results per term will be screened, supplemented by manual searches of relevant topic pages. One reviewer will conduct the screening, while a second reviewer will check 25%. Disagreements will be resolved through discussion. Data extraction will follow the same template as the scoping review, with additional source-specific fields (issuing body, document type, year, URL, and date).

Because this uses purposive sampling, extraction will continue until 2 consecutive sources yield no new candidate concepts or definitions, relative to those already charted. The source at which saturation was reached will be reported in the source log. Selected documents will be reported in the PRISMA-ScR flow diagram under records identified via methods other than database searching.

### Interoperability

To facilitate interoperability, WPV-ONTO will interface with current terminology and classification systems where appropriate. First, we will align WPV-ONTO principles with ICD-11 to facilitate integration with health documentation and reporting, particularly for categorizing health outcomes such as injuries and specific mental health concerns, as well as related conditions. Second, we will create a SNOMED CT mapping of related concepts to allow for semantic integration with electronic medical records and clinical workflows. This will be especially valuable when integrating WPV incident data to clinically relevant descriptors like injury and symptom concepts, as well as subsequent actions. Third, we will analyze ISLP alignment to allow cross-sector collaboration with courts and law enforcement. In addition, alignment with European occupational-injury statistics (European Statistics on Accidents at Work) and multilingual labeling, following Simple Knowledge Organization Systems conventions, will be presented to the expert panel as candidate requirements (Multimedia Appendix 3). When variations in purpose or legal frameworks impede direct mapping, we will identify the mismatch and develop a linkage layer that maintains semantic integrity while allowing for interoperable incident reporting across public services. Because linking to electronic health records and justice or police information systems entails highly sensitive personal data, WPV-ONTO is restricted to the ontology’s conceptual and terminological framework; it does not retain or transmit identifiable incident records. Any actual data linkage constructed on top of it must conform with applicable data protection legislation (eg, the EU General Data Protection Regulation) and adhere to data minimization and purpose limitation standards. Any pilot linkage between systems will necessitate its own ethical and data protection review, independent of the ontology-development activities detailed in this protocol.

### Evaluation

Consistent with METHONTOLOGY [30], WPV-ONTO will undergo iterative evaluation to ensure both verification and validation. These are treated as distinct questions: verification asks whether WPV-ONTO has been built correctly against its own stated requirements, while validation asks whether it is the right ontology for its intended users’ needs. The assessment will concentrate on semantic consistency, clear definitions, and comprehensive coverage of the WPV domain in public services. Verification during and after development will consist of consistency checks performed using HermiT, an open-source OWL reasoner [37].

Validation will combine 2 elements. Domain experts will carry out qualitative evaluations by developing competency questions that reflect real-world cross-sector reporting and follow-up scenarios. These questions will test whether WPV-ONTO accurately represents intended meanings and supports effective querying across key aggression subdomains identified during data charting. Feedback will be gathered through structured reviews and semistructured interviews focused on conceptual fit, ambiguity, and usability.

Once a prototype ontology-enabled form is available, we will conduct a small pilot usability evaluation with frontline workers. This feasibility study aims to identify usability issues and inform revisions before releasing WPV-ONTO (version 1.0). A broader evaluation, including interrater reliability testing, will occur in the implementation study described under the Future Directions section.

We will recruit 6 to 8 participants from health care and public transport, all currently employed in frontline roles involving public contact for at least 6 months, and who have used their organization’s incident reporting system. Members of the WPV-ONTO expert panel are excluded. Recruitment will be through sector organizations on the national committee on aggression against public-service workers. Participants will encode 8 incident vignettes using the prototype, constructed from anonymized descriptions reviewed by experts for realism and sectoral fidelity. The vignette set will be finalized before the pilot begins and published with the pilot results. Vignettes will cover act types, perpetrator relationships, and severity levels, including boundary cases like threat versus intimidation to test the ontology. If a sector’s reporting form is available through existing systems and code lists, participants will also encode vignettes on that form in a counterbalanced order to control for learning effects. Without a comparator form, the prototype is evaluated without a baseline. Two primary quantitative outcomes will be recorded: the proportion of vignettes encoded without a “miscellaneous” category and perceived usability, measured using the System Usability Scale (SUS). Completion time will be noted when a comparator form is available. A short semistructured interview will explore problems with applying concepts and perceived registration speed. Proportions will have 95% CIs, and SUS scores will be reported as means with SDs and medians. Interview data will be analyzed thematically and reported in accordance with COREQ (Consolidated Criteria for Reporting Qualitative Research) [38]. Quantitative results are descriptive, with no hypothesis testing. Interrater agreement is not assessed due to sample size constraints, but 2 usability criteria are set: a mean SUS score of at least 68 and fewer than 10% of vignettes using a residual category. Failure to meet these thresholds will prompt for revisions before the version 1.0 release, and documentation of missed criteria, revisions, and outcomes will be provided.

Expert validation and pilot findings will inform the governance process under the Maintenance and Further Development section, ensuring that evaluation results lead to traceable ontology changes. Each finding is logged as a change request and triaged by the domain editorial board (DEB) based on existing criteria. Terminological issues, like misinterpreted labels, lead to revised labels, definitions, or synonyms in the next minor release. Structural issues, such as unencodeable vignettes, result in new or restructured classes, properties, or relationships; those impacting downstream implementations are postponed to the next major release. Scope findings, with stated rationale, are deferred to later versions, and sector-specific findings go to the appropriate working group. All change requests and evaluation outcomes will be published with the version 1.0 release.

### Maintenance and Further Development

Consistent with the maintenance activity in METHONTOLOGY’s ontology development life-cycle [30], a staged, release-based governance model will be used by WPV-ONTO for maintenance and ongoing expansion (Figure 3). This strategy will separate less frequent structural upgrades from routine updates. The DEB, which oversees definitions, semantic coherence, and scope, will collaborate with the ontology engineering team, responsible for implementation, quality checks, and releases. When changes impact reporting practices specific to a sector, input from the sector’s working groups will be considered. A transparent change request system will be used to submit proposed additions (eg, new concepts, synonyms, definitional refinements, and mappings), which will then be reviewed for logical consistency, scope alignment, duplication, and approval. Major releases will occur once a year to accommodate restructuring, merging ideas, or model modifications that could affect downstream implementations, while scheduled minor releases will be published every 3 months and include incremental improvements, synonym or definition updates, and nonbreaking adjustments. To allow comparison of ideas over time, they will be marked as inactive, assigned stable IDs, and linked with explicit replacement references. Each release will include version numbers and brief change notes detailing additions, deprecations, merges, and definition modifications. This process will help researchers and implementers track progress while maintaining consistent cross-sector reporting.

**Figure 3:**
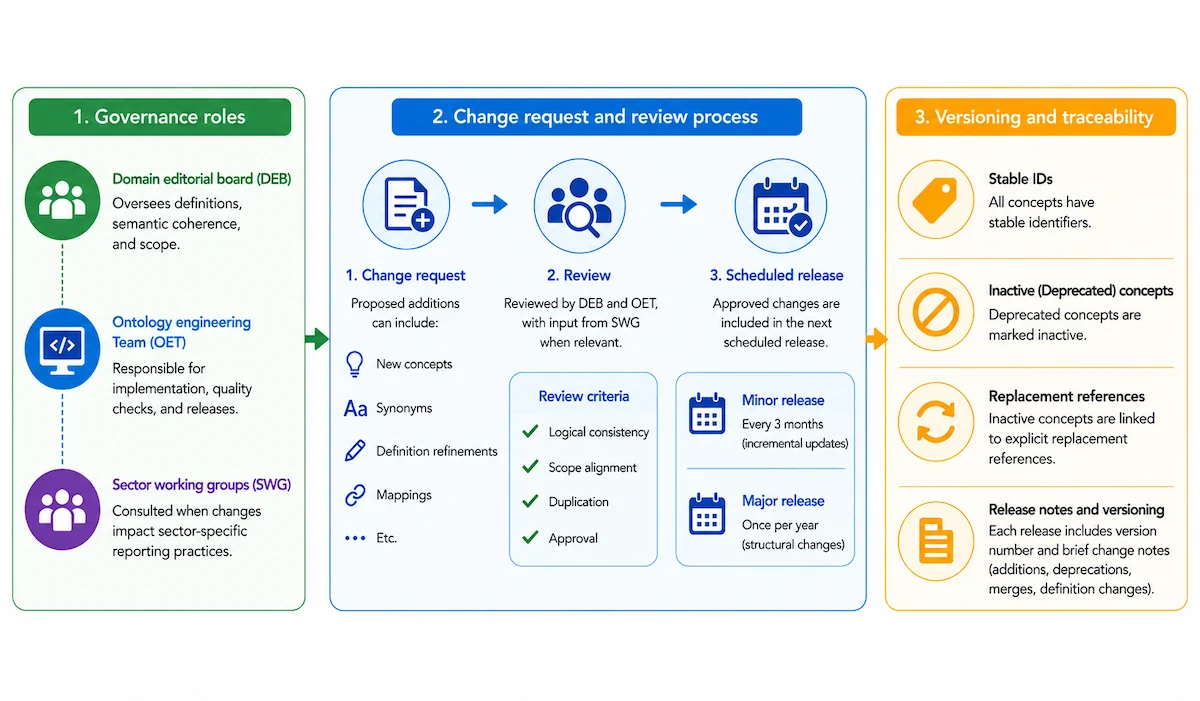
WPV-ONTO maintenance and release workflow: this governance model ensures transparent evolution, traceability of changes over time, and consistent and comparable cross-sector reporting for researchers and implementers. WPV-ONTO: workplace violence ontology.

### Reporting Standards

Because this protocol combines methodologically distinct components—a scoping review, ontology engineering, expert consensus, and a usability evaluation—no single checklist adequately captures the full study. The scoping review will be reported according to PRISMA-ScR [36], as described earlier. Ontology development and its documentation will follow the MIRO (Minimum Information for Reporting an Ontology) guidelines [39], which specify reporting of scope, competing resources, target audience, design rationale, source vocabularies, evaluation approach, and versioning policy. The expert-consensus and Delphi-informed decision rounds described under the Stakeholder Governance: Iterative Concept Refinement and Consensus Resolution section will be reported in line with the CREDES [31]. The usability evaluation, comprising competency-question testing and semistructured expert interviews, will be reported following the COREQ [38], insofar as it applies to interview-based data collection and analysis. Reporting against each of these frameworks will be prepared as supplementary material accompanying the resulting publications.

### Ethical Considerations

This paper describes a research protocol; no participants have been recruited, and none of the data-collection activities described here have yet taken place. Ethics approval for expert panel meetings, the Delphi-informed consensus rounds, and the pilot usability evaluation will be sought from the Ethics Committee for the Human Sciences at Vrije Universiteit Brussel before any of these activities begin. Prospective panel members and pilot participants will receive a participant information sheet (Multimedia Appendices 1 and 6) and will provide written informed consent (Multimedia Appendices 2 and 7) before participating. These separate documents reflect the different nature and extent of their involvement. Participant characteristics (eg, sector and years of relevant experience) will be collected and reported only in anonymized, aggregate form, and no directly identifying information will be published. Data will be stored and processed in accordance with the EU General Data Protection Regulation. No compensation is planned for participation in the stakeholder governance process or the pilot usability evaluation. The protocol will be registered on the Open Science Framework prior to the start of recruitment. The registration DOI will be reported in resulting publications.

## Results

As of July 2026, the project described in this protocol has not yet commenced: no participants have been recruited, and no data have been collected. The application to the Ethics Committee for the Human Sciences of the Vrije Universiteit Brussel will be submitted before recruitment of the expert panel begins. Recruitment is planned to start in March 2027, initiating the 24-month development cycle outlined in Figure 2. Iterative expert-consensus work will begin in month 3. The pilot usability evaluation is scheduled for months 18-22 of the development cycle, with findings incorporated before the version 1.0 release. WPV-ONTO (version 1.0) is expected at the end of the project period (anticipated early 2029).

WPV-ONTO will be developed using the outlined methodological framework and shared through established public-sector governance and scientific channels to ensure credibility and adoption. The research team is part of a national committee that addresses violence against public servants [40]. This committee includes representatives from key Belgian public entities such as the health sector, public transport, postal services, and security services. As a result, WPV-ONTO is likely to gain wider acceptance after its launch, thanks to a clearer mechanism for cross-sector consultation, adaptation to operational realities, and dissemination across stakeholder networks. WPV-ONTO will be officially registered as a reference nomenclature and, in a later phase, integrated into a planned generic cross-sector incident reporting system. This will enable structured, comparable reporting and interoperability across sectors, supporting future research, prevention, evaluation, and aftercare pathways.

## Discussion

This protocol is expected to result in a versioned, cross-sector WPV-ONTO for public services, together with an empirically grounded account of the shared and sector-specific concepts, definitions, and boundary ambiguities that currently prevent consistent incident registration. The discussion below situates this anticipated contribution relative to existing classification efforts, considers the strengths and limitations of the proposed approach, and outlines directions for future work and dissemination.

### Principal Findings

WPV-ONTO is anticipated to provide, for the first time, a consistent semantic language for describing WPV across public-service sectors that currently rely on incompatible, locally defined coding practices. While existing classification efforts capture WPV within single sectors, WPV-ONTO’s hierarchy of explicitly related concepts is expected to make incident descriptions comparable across sectors that have historically been studied in isolation, without discarding sector-specific nuance. If this expectation is realized, patterns that are currently invisible to sectors without a shared vocabulary, such as how exposure translates into response and outcome, may become visible. Because WPV-ONTO is intended to be interoperable with health and justice information systems, its cross-sector use is projected to extend beyond research to operational reporting and follow-up.

### Comparison to Prior Work

Sector-specific initiatives, including the California Division of Occupational Safety and Health’s type I-IV typology, the OSHA framework tailored for health care environments, and the UK’s Violent Incident Form, offer valuable organizational frameworks within their respective sectors. However, these frameworks were not designed for cross-sector comparability, and none have been defined as a machine-readable ontology. WPV-ONTO distinguishes itself from these initiatives by using a formal ontology-engineering methodology, mirroring the overarching logic used by Golec et al [32] to create a cross-disciplinary ontology for fibroproliferative diseases, rather than relying on a static, closed-list taxonomy. Existing general-purpose terminologies, such as ICD-11 and SNOMED CT, cover some relevant health outcomes; however, they were not specifically designed to capture the dynamics of perpetrator-target relationships, organizational contexts, and response pathways that are critical for registering WPV. WPV-ONTO strives to improve rather than copy these existing systems, by creating mappings where possible and introducing new structural features only where present systems lack sufficient granularity.

### Strengths and Limitations

A benefit of the proposed methodology lies in its integration of an iterative literature review, systematic extraction from industry code lists, and consensus among interdisciplinary experts. This is further reinforced by formal consistency verification through the HermiT reasoner and usability testing based on competency questions. This multisource, staged design aims to mitigate the risk of the ontology being influenced by the assumptions of any single sector. A notable advantage is the clearly defined versioning and governance model, which addresses the maintenance challenges that have constrained the long-term applicability of certain previous classification systems.

The source coverage for the targeted review of standards, guidance, and gray literature is purposive rather than comprehensive. The ontology’s scope is empirically tested through evaluation with competency questions and an encodability test, enabling additional concepts to be added later via the change-request process in the Maintenance and Further Development section.

A limitation is that semantic infrastructure alone cannot address the institutional and cultural factors that contribute to underreporting: treating a shared vocabulary as sufficient would risk technofetishism, in which technical infrastructure is expected to solve problems that have organizational and cultural roots. If institutions do not act on reports or if registration remains difficult, reporting rates may not increase merely because a shared vocabulary exists. This pattern has also been reported for certain WPV subtypes, such as sexual harassment, where data indicate that training and institutional follow-through, rather than documentation alone, are the primary constraints on disclosure and response [41,42]. The concern will be empirically investigated, but only preliminarily: the small-scale pilot usability evaluation is a feasibility study, and a full assessment of reporting burden reduction in practice requires the larger implementation study under the Future Directions section. WPV is also a contentious construct, and the distinctions between violence, incivility, harassment, and behavior that is considered “part of the job” reflect social and organizational norms that an ontology can express but cannot resolve. While iterative expert review and versioning are designed to keep WPV-ONTO responsive to these issues, ongoing stakeholder input will be essential to avoid definitional drift and the eventual loss of operational relevance.

A related limitation concerns terminology. Some terms used in this protocol, for consistency with existing literature and coding systems, including “perpetrator” and “noncompliant,” may read as stigmatizing or insufficiently trauma-informed, particularly in health care contexts where aggression can stem from a patient’s underlying condition rather than intent. WPV-ONTO’s own labels need not uncritically inherit this language: the expert-consensus process described under the Stakeholder Governance: Iterative Concept Refinement and Consensus Resolution section will explicitly revisit terminology choices, and we intend to seek input from patient or client advisors where feasible so that the final vocabulary is accurate without being stigmatizing.

An additional constraint is the protocol’s generalizability beyond the country environment in which it is based. Both the governance model and the national committee described in the Results section, as well as the ISLP mapping described in the Interoperability section, are based on Belgian structures; this supports feasibility but does not prove that WPV-ONTO seamlessly transfers to other countries’ legal and health-information systems. However, the ontology’s core structure is built around OWL-based concepts and mappings to international standards (ICD-11 and SNOMED CT) rather than Belgium-specific categories, and prevalence and risk-factor studies from other national contexts, such as China [14], describe WPV patterns broadly consistent with those motivating this protocol, indicating that the underlying conceptual structure may be portable even where country-specific governance and legal mappings would need adaptation. Directly testing this portability is beyond the scope of this protocol and is noted under the Future Directions section.

### Future Directions

This protocol is scoped to public services; extending WPV-ONTO’s conceptual framework to other sectors in later phases, as outlined in the Introduction section, will require testing whether its core structure generalizes beyond the public-service context or requires sector-specific extension. Future work should also evaluate WPV-ONTO empirically once it is integrated into reporting tools—for example, by assessing whether ontology-enabled forms measurably reduce reporting time or improve classification consistency relative to existing free-text or fixed-category systems and by tracking whether cross-sector comparisons enabled by WPV-ONTO reveal patterns not visible under current sector-specific registration. Given the Belgium-grounded governance model noted under the Strengths and Limitations section, future work should also test WPV-ONTO’s portability to other national legal and health-information systems, for example, through a collaboration with researchers in another country to assess whether the core concept structure needs revision or only its country-specific mappings (eg, to a non-Belgian police or justice system) need to be substituted.

More broadly, we believe that a well-developed ontology will enhance opportunities to move from isolated local registration to transparent, cross-sector knowledge, and that integrating it into specially designed reporting tools is one of its most immediately effective ways to promote practical adoption—a hope this protocol is designed to test empirically rather than assume.

### Dissemination Plan

Dissemination will target 3 audiences on different timelines: domain experts and the informatics community, through conference presentations and the peer-reviewed publication of the finalized ontology and protocol; public-service organizations and policymakers, through the research team’s involvement in a national committee on aggression against public-service workers and its constituent sectors (health, transport, postal, and security services); and software developers, through open publication of the OWL files and documentation needed to build ontology-enabled reporting tools. Major and minor releases will be announced through the same channels to support ongoing uptake as WPV-ONTO matures.

### Conclusions

This protocol outlines an evidence-based, expert-led approach for developing WPV-ONTO, the first cross-sector ontology for WPV in public services. The contribution this protocol seeks to establish is methodological: a staged, iteratively validated pathway from fragmented, sector-specific coding practices toward a shared, machine-readable vocabulary, together with a governance model intended to keep that vocabulary current and interoperable. Whether WPV-ONTO achieves the comparability and uptake it is designed for can only be established once it has been built and evaluated; this protocol does not itself demonstrate a reduction in underreporting or an improvement in worker outcomes. What it does offer is a transparent, replicable method by which such claims could subsequently be tested—for instance, by comparing incident registration before and after ontology-enabled reporting is introduced in a given sector. If successful, WPV-ONTO may, in time, provide public-service organizations, researchers, and policymakers with a common basis for cross-sector comparison and a foundation on which more consistent prevention, aftercare, and research efforts can be built.

## Data Availability

Data sharing is not applicable to this paper, as no datasets were generated or analyzed during this study.

## Appendix

Multimedia Appendix 1 Prospective panel members—participant information sheet.

Multimedia Appendix 2 Prospective panel members—informed consent sheet.

Multimedia Appendix 3 Delphi expert consultation: round 1 developing a cross-sector ontology for workplace violence in public services.

Multimedia Appendix 4 Search strategy: scientific databases.

Multimedia Appendix 5 Search strategy: targeted review of standards, guidance, and gray literature.

Multimedia Appendix 6 Pilot usability evaluation—participant information sheet.

Multimedia Appendix 7 Pilot usability evaluation—informed consent form.

## Abbreviations

COREQ: Consolidated Criteria for Reporting Qualitative Research
CREDES: Guidance on Conducting and Reporting Delphi Studies
DEB: domain editorial board
ICD-11: International Classification of Diseases, 11th Revision
ISLP: Integrated System for the Local Police
MIRO: Minimum Information for Reporting an Ontology
OSHA: Occupational Safety and Health Administration
OWL: Web Ontology Language
PICOS: population, intervention or exposure, comparator, outcome, study design
PRISMA-ScR: Preferred Reporting Items for Systematic Reviews and Meta-Analyses Extension for Scoping Reviews
SNOMED CT: Systematized Nomenclature of Medicine—Clinical Terms
SUS: System Usability Scale
WPV: workplace violence
WPV-ONTO: workplace violence ontology
