# Supplementary material for: An Ontology for Workplace Violence: Protocol to Support Cross-Sector Incident Reporting in Public Services
Source: JMIR Res Protoc. 2026 Oct 15;15:e94392. doi: 10.2196/94392 (PMC13720405; doi:10.2196/94392)
Supplement: Supplementary file 1 [file resprot_v15i1e94392_app1.docx]

**Study title:** An Ontology for Workplace Violence: Protocol to Support Cross-Sector Incident Reporting in Public Services

**Principal Investigator:** Iris Steenhout

**Contact:** [Iris.Steenhout@vub.be](mailto:Iris.Steenhout@vub.be) - +32 486386641

**Research Team:** REDM, Vrije Universiteit Brussel, Pleinlaan 2, 1050 Brussels

**Ethics reference:** to be completed

**DPO:** Vrije Universiteit Brussel, Pleinlaan 2 – 1050 Brussels, Belgium. Contact: dpo@vub.be

Dear colleague,

You are warmly invited to join the expert panel for WPV-ONTO, a research initiative to develop the first open-access, cross-sector ontology and reference nomenclature for workplace violence (WPV) in public services. Your specialist knowledge makes your perspective particularly valuable to this work.

Workplace violence, encompassing physical assault, verbal aggression, threats, harassment, and related hostile acts against workers, is one of the most prevalent occupational hazards across public-service sectors worldwide. Despite this, a major obstacle to coordinated prevention and learning remains: incidents are described and coded differently across organizations, sectors, and countries, making comparison and cumulative understanding effectively impossible. WPV-ONTO aims to remedy this by providing a shared, semantically rich vocabulary that works across healthcare, education, social welfare, policing, emergency response, and public transport.

This Participant Information Sheet sets out everything you need to know about the study. Please read it carefully. You are under no obligation to participate, and you may take as much time as you need to decide. If you have any questions, please contact the principal investigator using the details above.

**Section 1: Purpose and Background**

**Why is this study being conducted?**

Underreporting is a defining problem in WPV registration across public services. This is compounded by inconsistent incident descriptions, the absence of a shared nomenclature, and the tendency to study WPV within sector-specific silos rather than through a common framework. Without a shared semantic structure, similar events are recorded differently across settings or remain uncodable and effectively invisible to researchers and policymakers.

WPV-ONTO addresses this by creating and openly publishing a formal ontology, a structured, machine-readable vocabulary, that captures WPV concepts, definitions, relationships, and synonyms relevant across public-service sectors.

**What will WPV-ONTO be used for?**

The completed ontology will serve as a public infrastructure resource to support:

- Cross-sector harmonization of incident reporting systems
- Software, API, and data integration development
- Research data interoperability and meta-analysis
- Evaluation of WPV prevention and aftercare programs
- Linkage with electronic health records, justice systems, and police information systems

**Section 2: Who We Are Looking For**

We are recruiting a heterogeneous expert panel of at least 30 participants. Diversity within the panel, across sectors, professional roles, geographic contexts, and methodological backgrounds, is essential to the quality and generalisability of WPV-ONTO. You are eligible to participate if you have relevant expertise in one or more of the following areas:

**Professional or frontline experience in public services**

Healthcare (nursing, medicine, emergency care, allied health)

Education (teaching, educational support, school/university administration)

Policing, law enforcement, or justice services

Social welfare, social work, or residential care

Emergency response (fire service, ambulance, civil protection)

Public transport (rail, bus, metro operations)

**Research, policy, or technical expertise**

Workplace violence research, epidemiology, or occupational health

Occupational safety, risk management, or aftercare provision

Ontology engineering, knowledge representation, or semantic technologies

Health informatics, information systems, or software development

WPV-related policy design, prevention strategy, or incident reporting governance

Victim advocacy or staff support in public-service environments

Legal expertise in data protection, privacy law, surveillance regulation, or linked/integrated database governance (e.g., cross-sector data sharing, secondary use of administrative data, police and justice information systems)

Participants who do not hold formal research roles but have substantial practical, frontline, or lived professional experience are equally encouraged to participate. The study team will recruit through professional networks, academic literature contacts, sector organizations, and social media.

**Section 3: What Does Participation Involve?**

**Overview of the process**

The study uses a modified Delphi technique, a structured, iterative consensus method widely used in health, social science, and information systems research. Expert panellists respond to questionnaires in multiple rounds, receive anonymized summaries of the panel's collective responses between rounds, and have the opportunity to revise their answers in light of group feedback. This approach prevents dominant voices from skewing results while allowing genuine expert deliberation. All questionnaires will be administered online via a secure survey platform. You may complete each round at a time that suits you within the given window. In addition to the survey rounds, panel members may be invited to periodic (online) project meetings; attendance is optional.

**Study timeline**

| **Phase** | **Time** | **What you will be asked to do** |
| --- | --- | --- |
| **Round 1** | ~30–45 min | Initial questionnaire: rate, comment on proposed WPV-ONTO concepts and definitions |
| **Feedback report** |  | Anonymized aggregate results shared with all panel members |
| **Round 2** | ~30–45 min | Revised questionnaire: re-rate items that did not reach consensus; new items suggested by the panel |
| **Feedback report** |  | Anonymized aggregate results of Round 2 shared with all panel members |
| **Round 3 (if needed)** | ~20–30 min | Final consensus round on any remaining unresolved items |
| **Results** |  | Copy of the final consensus report and any resulting publications sent to participants |

**What will the questionnaires cover?**

Each consultation round will ask you to review proposed WPV-ONTO content across three thematic clusters:

**1. Conceptual and definitional scope**

- Proposed definitions of WPV and its constituent types (physical assault, verbal aggression, threats, harassment, bullying, discrimination-motivated violence, etc.)
- Proposed boundaries between WPV and adjacent concepts (occupational stress, burnout, organizational conflict)
- Synonyms, cross-language equivalents, and sector-specific terminology

**2. Ontological structure and relationships**

- Proposed hierarchical classes and subclasses within WPV-ONTO
- Object properties (e.g., 'directed at', 'perpetrated by', 'occurs in context of', 'has consequence')
- Data properties and annotation fields relevant to incident encoding
- Inheritance and compositional encoding rules

**3. Cross-sector applicability and reporting practice**

- Fit of proposed concepts with sector-specific reporting cultures and tools
- Gaps or omissions identified from your professional experience
- Priority features for integration with existing information systems (EMR, justice databases, HR systems)
- Recommendations for version governance and ongoing maintenance of WPV-ONTO

For each item you will be asked to rate your level of agreement or priority using a 7-point Likert scale, and you will have the option to provide free-text comments explaining your rating or suggesting revisions. Consensus will be defined as at least 75% of panellists rating an item 5 or higher on the 7-point scale.

**Section 4: Voluntary Participation and Withdrawal**

Participation is entirely voluntary. You are free to:

- Decline to answer any individual question within a questionnaire
- Skip a round without withdrawing from the study altogether
- Withdraw from the study at any time, without giving a reason, and without any negative consequence

To withdraw, simply email the principal investigator at the address listed at the top of this document. If you withdraw before the final dataset is locked, you may also request that your data be deleted. If your anonymized responses have already been incorporated into aggregate results shared with other panel members, full retrospective deletion may not be possible; the team will advise you on what is feasible at the time of your withdrawal.

**Section 5: Potential Benefits and Risks**

**Benefits**

There is no direct personal benefit to you from participating. However, your contribution will help shape an open-access resource expected to improve how workplace violence is understood, reported, and prevented across public-service sectors. Panellists will receive:

- A copy of the anonymized aggregate results after each round
- A copy of the final consensus report
- An acknowledgment of their expert contribution in any resulting publications (unless you prefer to remain unacknowledged)
- Early access to WPV-ONTO and associated publications

**Risks**

The risks associated with this study are minimal. The questionnaires do not ask about personal experiences of victimization. However, because the subject matter concerns workplace violence, some participants may find certain conceptual discussions or scenario descriptions sensitising. If you find any content distressing at any point, you are free to skip questions or pause and return to the survey, and you may withdraw without penalty.

Should you wish to access occupational mental health support, we encourage you to consult your employer's employee assistance programme or an occupational health service. A list of general support resources is provided at the end of this document.

**Section 6 — Confidentiality and Anonymity**

The Delphi method is designed to prevent dominant voices from distorting group opinion. To this end:

- Individual responses are never shared with other panel members - only anonymized aggregate results are circulated between rounds
- No panellist will know the identities of other participants unless they disclose this themselves
- Any direct quotes included in publications will be reported without attribution
- Participation lists will not be published

Contact details (name and email) are held separately from questionnaire responses and will not appear in any dataset used for analysis or publication. All data are stored on encrypted, password-protected institutional servers accessible only to named members of the research team.

**Section 7 — How Your Personal Data Will Be Used (GDPR)**

This study is conducted in accordance with Regulation (EU) 2016/679, the General Data Protection Regulation (GDPR).

**What personal data are collected?**

- Professional background (sector, role, years of experience, country): collected in Round 1 to characterize the panel
- Contact details (name, email address): used only to send survey links, reminders, and result summaries
- Questionnaire responses: Likert-scale ratings and free-text comments

No special-category personal data are collected. You will not be asked about your health, ethnicity, religion, political opinions, or personal experiences of victimization.

**Legal basis for processing**

**Consent:** the primary basis for processing your contact details and questionnaire responses.

**Public interest / scientific research:** provides a supplementary basis for processing anonymized research data in the public interest, in conjunction with applicable national implementing legislation.

**How long will your data be kept?**

Contact details and individually identifiable responses will be retained for a maximum of five years from the end of the project, in line with good scientific practice guidelines and applicable institutional data retention policy. Anonymized data integrated into WPV-ONTO or incorporated into publications will be retained indefinitely as part of the scientific record.

**Will your data be transferred outside the EU/EEA?**

Data are processed within the European Economic Area (EEA).

**Your rights under GDPR**

You may exercise any of the following rights at any time by contacting the Data Controller or DPO:

| **Your right** | **What it means in practice** |
| --- | --- |
| **Right of access** | Request confirmation that your data are processed and obtain a copy. |
| **Right to rectification** | Ask us to correct inaccurate or incomplete personal data. |
| **Right to erasure** | Request deletion of your data, subject to research exemptions under Art. 17(3)(d). |
| **Right to restriction** | Ask us to limit how your data are used while a dispute is resolved. |
| **Right to data portability** | Receive your data in a structured, machine-readable format. |
| **Right to object** | Object to processing based on public interest or legitimate interests. |
| **Right to withdraw consent** | Withdraw consent at any time without affecting the lawfulness of prior processing. |

You also have the right to lodge a complaint with a data protection supervisory authority. In Belgium, this is:

**Gegevensbeschermingsautoriteit / Autorité de protection des données (GBA/APD)**

Rue de la Presse 35 / Drukpersstraat 35, 1000 Brussels

www.gegevensbeschermingsautoriteit.be | contact@apd-gba.be | +32 2 274 48 00

*You may also contact the supervisory authority in the EU Member State of your habitual residence or place of work.*

**Section 8 — Results and Dissemination**

The results of this study will be disseminated in the following ways:

- A final WPV-ONTO consensus report will be shared with all participating panel members
- Research findings will be submitted for publication in peer-reviewed journals and/or presented at relevant conferences
- Panel members will receive copies of any publications resulting from this work

No individually identifiable information will appear in any publication. Anonymized quotes may be reported to illustrate expert opinion; these will not be traceable to individual panellists.

**Section 9: How to Express Your Interest**

If you are willing to participate, please express your interest by contacting the principal investigator using the details at the top of this document. You will then receive a link to the online consent form and, once your consent is registered, the first-round questionnaire.

You are also warmly encouraged to share this invitation with colleagues who may meet the inclusion criteria. A broader and more diverse panel strengthens the representativeness and authority of WPV-ONTO.

If you do not wish to participate, no further action is needed. Please simply disregard this invitation.

Thank you sincerely for your time and consideration. We hope you will join us in this important work.

**Complaints and concerns**

If you have any concerns about the conduct of this research that you do not feel comfortable raising with the research team, please contact the Ethics Committee for the Human Sciences of the Vrije Universiteit Brussel: [echw@vub.be](mailto:echw@vub.be).

**Support Resources**

While this study does not address personal experiences of victimization, the following organizations offer support related to workplace wellbeing and violence:

| **Organization / resource** | **Contact** |
| --- | --- |
| **European Agency for Safety and Health at Work (EU-OSHA)** | osha.europa.eu |
| **PREVENT (Belgian prevention institute)** | www.prevent.be |
| **Employee Assistance Programme (EAP)** | Contact your employer's HR or occupational health service |
| **Centre de prévention des violences conjugales et familiales (BE)** | www.cpvcf.org |
| **Your occupational health / wellbeing service** | Contact your employer |
