# Supplementary material for: An Ontology for Workplace Violence: Protocol to Support Cross-Sector Incident Reporting in Public Services
Source: JMIR Res Protoc. 2026 Oct 15;15:e94392. doi: 10.2196/94392 (PMC13720405; doi:10.2196/94392)
Supplement: Supplementary file 2 [file resprot_v15i1e94392_app2.docx]

**Study title:** An Ontology for Workplace Violence: Protocol to Support Cross-Sector Incident Reporting in Public Services

**Principal Investigator:** Iris Steenhout

**Ethics reference:** to be completed

You are being invited to participate as an expert in a research study aimed at developing WPV-ONTO, a shared, open-access ontology and reference nomenclature for workplace violence (WPV) across public-service sectors. This study is being conducted in accordance with the ethical principles of the Declaration of Helsinki and applicable European Union (EU) legislation, including Regulation (EU) 2016/679 (the General Data Protection Regulation, GDPR).

**Participation is entirely voluntary. You may withdraw your consent at any time, without giving a reason, and without any negative consequences.** To withdraw, simply contact the principal investigator using the details in the information sheet. Data already collected prior to your withdrawal will be retained unless you specifically request otherwise.

**Part A: About the Study**

**1. Purpose and background**

Workplace violence (WPV), encompassing physical assault, verbal aggression, threats, and harassment directed at workers, is widespread and chronically underreported across public services, including healthcare, education, social welfare, law enforcement, emergency response, and public transport. A core obstacle to coordinated learning and prevention is the absence of a shared vocabulary: similar incidents are described and coded differently across organizations and sectors, making comparison and cumulative analysis difficult. WPV-ONTO will address this gap by producing a versioned, available ontology.

**2. Why you have been invited**

You have been identified as a domain expert in one or more of the following areas:

- Frontline public-service work (healthcare, education, policing, transport, social welfare, or emergency response)
- Occupational health, safety, or aftercare in public services
- Workplace violence research or epidemiology
- Semantic technologies, ontology engineering, or health/information systems
- Sectoral policy, prevention strategy, or incident reporting governance
- Lived or professional experience of WPV reporting and registration
- Legal expert with expertise in privacy, (linked) databases and surveillance

**3. What participation involves**

If you agree to take part, you will be asked to contribute to one or more structured expert consultation rounds. Each round consists of an online questionnaire covering proposed WPV concepts, definitions, relationships, and nomenclature. You will be invited to rate, comment, and suggest revisions. You will receive anonymized summaries of the panel's aggregate responses between rounds so that you can reconsider your inputs in light of collective expert opinion.

**Estimated time commitment:** approximately 30–45 minutes per consultation round. A maximum of three rounds are planned.

# **Part B: Data Protection (GDPR)**

In accordance with Regulation (EU) 2016/679 (GDPR), we inform you of the following.

**4. Data controller**

Vrije Universiteit Brussel, Pleinlaan 2 – 1050 Brussels, Belgium. Contact: dpo@vub.be

**5. Categories of personal data collected**

- Professional background information (sector of expertise, professional role, years of experience)
- Opinions, ratings, and free-text comments submitted in the consultation rounds
- Contact details (name and email address), used solely to send survey links, reminders, and result summaries

No special-category personal data, such as health, ethnicity, or political opinions, are collected. You will not be asked to disclose personal experiences of victimization.

**6. Purpose and use of your data**

Your data will be used exclusively for:

- Conducting the expert consultation rounds and generating aggregate consensus outputs
- Producing anonymized summary reports shared with panel members between rounds
- Publishing research findings in peer-reviewed journals and conference proceedings
- Developing and refining WPV-ONTO as a public open-access resource

No individually identifiable data will be published. Anonymized quotes may be reported; these will not identify you.

**7. Data recipients and transfers**

Your data will be accessible only to members of the core research team bound by confidentiality obligations. Anonymized aggregate results will be shared with the full expert panel. No personally identifiable data will be shared with third parties. Qualtrics, the online survey platform used processes data within the European Economic Area (EEA).

**8. Retention period**

Contact details and individual responses will be retained for a maximum of five years from the end of the project, in accordance with applicable institutional data management policies and good scientific practice guidelines. Anonymized data integrated into WPV-ONTO or published outputs will be retained indefinitely as part of the scientific record.

**9. Your rights as a data subject**

Under GDPR, you have the following rights, which you may exercise at any time by contacting the Data Controller:

- Right of access: to obtain confirmation of whether your data are processed and to receive a copy
- Right to rectification: to have inaccurate data corrected
- Right to erasure: to request deletion of your data, subject to legal research exemptions
- Right to restriction of processing: to request that processing be limited
- Right to data portability: to receive your data in a structured, machine-readable format
- Right to object: to object to processing based on legitimate interests or public interest
- Right to withdraw consent: at any time, without affecting the lawfulness of prior processing

You also have the right to lodge a complaint with a supervisory authority. In Belgium, this is the Gegevensbeschermingsautoriteit / Autorité de protection des données (GBA/APD): www.gegevensbeschermingsautoriteit.be. You may also contact the supervisory authority in the EU Member State of your usual residence or place of work.

# **Part C: Declaration of Consent**

Please read each statement carefully and place a tick (✓) in the checkbox to indicate that you agree. You must tick all items to participate.

| ☐ | **1.** I confirm that I have read and understood the Participant Information Sheet (version [X], dated [date]) for the WPV-ONTO study and have had the opportunity to ask questions and receive satisfactory answers. |
| --- | --- |
| ☐ | **2.** I understand that my participation is entirely voluntary and that I may withdraw at any time, without giving a reason, and without any negative consequences. |
| ☐ | **3.** I understand that my individual responses and contact details will be kept strictly confidential and accessible only to the research team. Other panel members will not be able to identify me. |
| ☐ | **4.** I consent to my anonymised opinions and quotes being used in research publications, the development of WPV-ONTO, and related scientific outputs. No information that could identify me will be published without my explicit separate consent. |
| ☐ | **5.** I understand that my personal data will be processed in accordance with the General Data Protection Regulation (GDPR) for the purposes described in this form, and I consent to this processing.  *Note: You may withdraw this consent at any time by contacting the principal investigator. Withdrawal does not affect the lawfulness of processing prior to withdrawal.* |
| ☐ | **6.** I am aware of my rights under GDPR, including the right to access, rectify, erase, or restrict my data, and know how to exercise these rights as described in Part B of this form. |
| ☐ | **7.** I understand that I may receive up to two email reminders per consultation round and a summary report between rounds. I consent to being contacted for these purposes. |
| ☐ | **8.** I agree to take part in the WPV-ONTO expert consultation study. |

**Signature**

| **Full name:** |  |
| --- | --- |
|  |  |
| **Signature:** |  |
|  |  |
| **Date:** |  |
|  |  |
| **Professional role / sector:** |  |

*This form has been prepared in accordance with Regulation (EU) 2016/679 (GDPR) and the Declaration of Helsinki. Please retain a copy of this signed form for your records. A copy will also be held securely for the duration of the project and for five years thereafter by the research team.*
