# Supplementary material for: An Ontology for Workplace Violence: Protocol to Support Cross-Sector Incident Reporting in Public Services
Source: JMIR Res Protoc. 2026 Oct 15;15:e94392. doi: 10.2196/94392 (PMC13720405; doi:10.2196/94392)
Supplement: Supplementary file 3 [file resprot_v15i1e94392_app3.docx]

Thank you for agreeing to participate in this Delphi survey.

This questionnaire is the first of up to three rounds of expert consultation. Its purpose is to reach consensus on the key concepts, definitions, structural features, and cross-sector requirements that should be incorporated into WPV-ONTO, an open-access, cross-sector ontology for workplace violence (WPV) in public services.

The questionnaire is organised into three thematic sections totalling 21 questions. It should take approximately 30–45 minutes to complete. You may work through it at your own pace and save your progress before submitting.

For each question, please rate how much or how little you would prioritise the inclusion of the proposed feature in WPV-ONTO, using the 7-point scale provided. There are no right or wrong answers: it is your expert opinion that matters. You are also warmly invited to elaborate on your rating and add comments in the open text fields below each question.

| ☐ | Tick the box that best represents your rating for each question. |
| --- | --- |
| ✎ | Use the open text fields to explain your rating or add suggestions. These fields are optional but greatly help the research team interpret results. |
| → | At the end of the questionnaire you will have the opportunity to suggest new questions for Round 2. |
| ⚑ | If any content is distressing, you may skip questions. Support resources are listed in your Participant Information Sheet. |

**Response deadline:** [ (2 weeks after distribution)]

Questions? Contact: Iris Steenhout – Iris.Steenhout@vub.be

**THEME 1**

Conceptual and Definitional Scope

Questions 1–7 What WPV-ONTO should define, include, and exclude

The questions in this section concern the core concepts and definitional boundaries of WPV-ONTO. For each question, please rate how much you would prioritise including the proposed feature in the ontology.

| Q01 | An explicit, formal definition of workplace violence (WPV) as a top-level class, covering all intentional harmful acts directed at a worker in the course of their duties, regardless of sector or perpetrator.  Note: This would serve as the root concept from which all other WPV types inherit. |
| --- | --- |

WPV-ONTO should prioritise:

| 1 | 2 | 3 | 4 | 5 | 6 | 7 |
| --- | --- | --- | --- | --- | --- | --- |
| Not a  priority | Low  priority | Somewhat  a priority | Neutral | Moderate  priority | High  priority | Essential  priority |
| ☐ | ☐ | ☐ | ☐ | ☐ | ☐ | ☐ |

Why did you choose this rating? (optional)

Additional comments or suggestions (optional)

| Q02 | Formal subclasses for each major type of WPV, including: physical assault or aggression; verbal aggression (shouting, insults, demeaning language); threats (explicit or implicit); harassment (including sexual harassment); bullying or mobbing; discrimination-motivated violence; and property damage directed at a worker.  Note: Consider whether all subtypes listed are conceptually distinct enough to warrant separate classes. |
| --- | --- |

WPV-ONTO should prioritise:

| 1 | 2 | 3 | 4 | 5 | 6 | 7 |
| --- | --- | --- | --- | --- | --- | --- |
| Not a  priority | Low  priority | Somewhat  a priority | Neutral | Moderate  priority | High  priority | Essential  priority |
| ☐ | ☐ | ☐ | ☐ | ☐ | ☐ | ☐ |

Why did you choose this rating? (optional)

Additional comments or suggestions (optional)

| Q03 | Explicit modelling of the perpetrator dimension, including: patient- or client-initiated violence; colleague- or co-worker-initiated violence (internal WPV); third-party violence (bystanders, family members, members of the public); and unknown/unidentified perpetrators.  Note: The perpetrator type is a key axis for sector-specific reporting and prevention strategy. |
| --- | --- |

WPV-ONTO should prioritise:

| 1 | 2 | 3 | 4 | 5 | 6 | 7 |
| --- | --- | --- | --- | --- | --- | --- |
| Not a  priority | Low  priority | Somewhat  a priority | Neutral | Moderate  priority | High  priority | Essential  priority |
| ☐ | ☐ | ☐ | ☐ | ☐ | ☐ | ☐ |

Why did you choose this rating? (optional)

Additional comments or suggestions (optional)

| Q04 | Explicit modelling of incident context and setting, including: physical location (ward, classroom, vehicle, outdoor public space, etc.); work schedule context (lone working, night shift, high patient/client load); and triggering circumstances (intoxication, mental health crisis, frustration with service). |
| --- | --- |

WPV-ONTO should prioritise:

| 1 | 2 | 3 | 4 | 5 | 6 | 7 |
| --- | --- | --- | --- | --- | --- | --- |
| Not a  priority | Low  priority | Somewhat  a priority | Neutral | Moderate  priority | High  priority | Essential  priority |
| ☐ | ☐ | ☐ | ☐ | ☐ | ☐ | ☐ |

Why did you choose this rating? (optional)

Additional comments or suggestions (optional)

| Q05 | Clear formal boundaries distinguishing WPV from adjacent concepts, specifically: occupational stress and burnout (not included as WPV); organisational conflict between colleagues (partially included where it meets a severity threshold); and incivility or rudeness (included only above a defined threshold of intent or impact).  Note: Vague boundaries are a primary cause of underreporting and inconsistent coding. |
| --- | --- |

WPV-ONTO should prioritise:

| 1 | 2 | 3 | 4 | 5 | 6 | 7 |
| --- | --- | --- | --- | --- | --- | --- |
| Not a  priority | Low  priority | Somewhat  a priority | Neutral | Moderate  priority | High  priority | Essential  priority |
| ☐ | ☐ | ☐ | ☐ | ☐ | ☐ | ☐ |

Why did you choose this rating? (optional)

Additional comments or suggestions (optional)

| Q06 | A standardised severity classification scheme within WPV-ONTO, capturing: physical consequences (no injury, minor injury, serious injury, fatality); psychological impact (transient distress, acute stress response, PTSD-level impact); and operational impact on the service (no disruption, temporary disruption, service interruption). |
| --- | --- |

WPV-ONTO should prioritise:

| 1 | 2 | 3 | 4 | 5 | 6 | 7 |
| --- | --- | --- | --- | --- | --- | --- |
| Not a  priority | Low  priority | Somewhat  a priority | Neutral | Moderate  priority | High  priority | Essential  priority |
| ☐ | ☐ | ☐ | ☐ | ☐ | ☐ | ☐ |

Why did you choose this rating? (optional)

Additional comments or suggestions (optional)

| Q07 | Systematic inclusion of synonyms, near-synonyms, and sector-specific terminology for each WPV class, for example: 'aggression' (healthcare), 'antisocial behaviour' (transport), 'assault' (policing/justice), 'targeted harassment' (education), to enable cross-sector mapping without loss of specificity. |
| --- | --- |

WPV-ONTO should prioritise:

| 1 | 2 | 3 | 4 | 5 | 6 | 7 |
| --- | --- | --- | --- | --- | --- | --- |
| Not a  priority | Low  priority | Somewhat  a priority | Neutral | Moderate  priority | High  priority | Essential  priority |
| ☐ | ☐ | ☐ | ☐ | ☐ | ☐ | ☐ |

Why did you choose this rating? (optional)

Additional comments or suggestions (optional)

**THEME 2**

Ontological Structure and Relationships

Questions 8–14 How WPV-ONTO should be structured, encoded, and governed

The questions in this section concern the internal architecture of WPV-ONTO — its class hierarchy, properties, relationships, and governance. Please rate how much you would prioritise each structural feature.

| Q08 | A strict hierarchical (subsumption) class structure, in which every WPV subtype inherits the properties of its parent class, enabling automated reasoning and inference across the ontology.  Note: This is a foundational design choice affecting how the ontology supports software reasoning and data integration. |
| --- | --- |

WPV-ONTO should prioritise:

| 1 | 2 | 3 | 4 | 5 | 6 | 7 |
| --- | --- | --- | --- | --- | --- | --- |
| Not a  priority | Low  priority | Somewhat  a priority | Neutral | Moderate  priority | High  priority | Essential  priority |
| ☐ | ☐ | ☐ | ☐ | ☐ | ☐ | ☐ |

Why did you choose this rating? (optional)

Additional comments or suggestions (optional)

| Q09 | A set of object properties (relations between classes) capturing: 'directed at' (links incident to worker/role); 'perpetrated by' (links incident to perpetrator class); 'occurs in' (links incident to setting); 'has consequence' (links incident to outcome class); and 'reported via' (links incident to reporting mechanism). |
| --- | --- |

WPV-ONTO should prioritise:

| 1 | 2 | 3 | 4 | 5 | 6 | 7 |
| --- | --- | --- | --- | --- | --- | --- |
| Not a  priority | Low  priority | Somewhat  a priority | Neutral | Moderate  priority | High  priority | Essential  priority |
| ☐ | ☐ | ☐ | ☐ | ☐ | ☐ | ☐ |

Why did you choose this rating? (optional)

Additional comments or suggestions (optional)

| Q10 | A set of data properties (attributes attached to instances) enabling structured incident encoding, including: date and time; recurrence flag (isolated/repeated); duration or frequency; formal report filed (yes/no/partial); and aftercare accessed (yes/no/type). |
| --- | --- |

WPV-ONTO should prioritise:

| 1 | 2 | 3 | 4 | 5 | 6 | 7 |
| --- | --- | --- | --- | --- | --- | --- |
| Not a  priority | Low  priority | Somewhat  a priority | Neutral | Moderate  priority | High  priority | Essential  priority |
| ☐ | ☐ | ☐ | ☐ | ☐ | ☐ | ☐ |

Why did you choose this rating? (optional)

Additional comments or suggestions (optional)

| Q11 | Explicit modelling of reporting and registration events as first-class entities within WPV-ONTO, including: the act of reporting, the reporting tool or system used, the reporting outcome (acted upon/dismissed/pending), and barriers to reporting (fear of reprisal, lack of awareness, perceived futility).  Note: Underreporting is the central problem this ontology addresses; modelling reporting events explicitly is one way to support its study. |
| --- | --- |

WPV-ONTO should prioritise:

| 1 | 2 | 3 | 4 | 5 | 6 | 7 |
| --- | --- | --- | --- | --- | --- | --- |
| Not a  priority | Low  priority | Somewhat  a priority | Neutral | Moderate  priority | High  priority | Essential  priority |
| ☐ | ☐ | ☐ | ☐ | ☐ | ☐ | ☐ |

Why did you choose this rating? (optional)

Additional comments or suggestions (optional)

| Q12 | A formal consequence and aftercare module capturing: short-term consequences (sick leave, reassignment, altered working practices); long-term consequences (occupational exit, chronic health impact, legal proceedings); and aftercare provision received (psychological support, legal advice, peer support, no aftercare). |
| --- | --- |

WPV-ONTO should prioritise:

| 1 | 2 | 3 | 4 | 5 | 6 | 7 |
| --- | --- | --- | --- | --- | --- | --- |
| Not a  priority | Low  priority | Somewhat  a priority | Neutral | Moderate  priority | High  priority | Essential  priority |
| ☐ | ☐ | ☐ | ☐ | ☐ | ☐ | ☐ |

Why did you choose this rating? (optional)

Additional comments or suggestions (optional)

| Q13 | Formal annotation properties on each class and property within WPV-ONTO, including: a human-readable definition; a rationale or scope note; at least one literature reference; and the ontology version in which the class was introduced or last modified.  Note: Annotation properties are essential for transparency, trustworthiness, and maintainability. |
| --- | --- |

WPV-ONTO should prioritise:

| 1 | 2 | 3 | 4 | 5 | 6 | 7 |
| --- | --- | --- | --- | --- | --- | --- |
| Not a  priority | Low  priority | Somewhat  a priority | Neutral | Moderate  priority | High  priority | Essential  priority |
| ☐ | ☐ | ☐ | ☐ | ☐ | ☐ | ☐ |

Why did you choose this rating? (optional)

Additional comments or suggestions (optional)

| Q14 | A versioned governance model for WPV-ONTO, including: a published changelog; a defined process for proposing and validating updates; and designated domain-expert reviewers for each sector covered by the ontology.  Note: Without version governance, an ontology becomes rapidly outdated and unreliable as a shared reference. |
| --- | --- |

WPV-ONTO should prioritise:

| 1 | 2 | 3 | 4 | 5 | 6 | 7 |
| --- | --- | --- | --- | --- | --- | --- |
| Not a  priority | Low  priority | Somewhat  a priority | Neutral | Moderate  priority | High  priority | Essential  priority |
| ☐ | ☐ | ☐ | ☐ | ☐ | ☐ | ☐ |

Why did you choose this rating? (optional)

Additional comments or suggestions (optional)

**THEME 3**

Cross-Sector Applicability and Reporting Practice

Questions 15–21 Fit with sector needs, information systems, law, and governance

The questions in this section concern how WPV-ONTO should function across public-service sectors and integrate with existing reporting tools, legal frameworks, and information systems. Please rate how much you would prioritise each feature.

| Q15 | A modular sector extension mechanism allowing core WPV-ONTO classes to be supplemented with sector-specific concepts without breaking the shared top-level structure, enabling healthcare, education, policing, transport, social welfare, and emergency services to maintain contextual specificity while retaining cross-sector comparability. |
| --- | --- |

WPV-ONTO should prioritise:

| 1 | 2 | 3 | 4 | 5 | 6 | 7 |
| --- | --- | --- | --- | --- | --- | --- |
| Not a  priority | Low  priority | Somewhat  a priority | Neutral | Moderate  priority | High  priority | Essential  priority |
| ☐ | ☐ | ☐ | ☐ | ☐ | ☐ | ☐ |

Why did you choose this rating? (optional)

Additional comments or suggestions (optional)

| Q16 | A formal mapping layer between WPV-ONTO classes and existing industry-standard reporting code lists and classification systems, such as: ICD-11 external cause codes; Eurostat’s ESAW (European Statistics on Accidents at Work) categories; national sector-specific incident forms; and police and justice classification codes.  Note: Without mapping to existing systems, adoption will be limited. However, overfitting to existing systems risks perpetuating their known deficiencies. |
| --- | --- |

WPV-ONTO should prioritise:

| 1 | 2 | 3 | 4 | 5 | 6 | 7 |
| --- | --- | --- | --- | --- | --- | --- |
| Not a  priority | Low  priority | Somewhat  a priority | Neutral | Moderate  priority | High  priority | Essential  priority |
| ☐ | ☐ | ☐ | ☐ | ☐ | ☐ | ☐ |

Why did you choose this rating? (optional)

Additional comments or suggestions (optional)

| Q17 | Explicit modelling of legal and regulatory concepts within WPV-ONTO, including: the distinction between acts that are criminal offences and those addressed through occupational/employment law; relevant EU legislative frameworks (e.g., OSH Framework Directive, GDPR where applicable to reporting data); and the concept of duty of care.  Note: Legal experts on the panel are asked particularly to consider this question. |
| --- | --- |

WPV-ONTO should prioritise:

| 1 | 2 | 3 | 4 | 5 | 6 | 7 |
| --- | --- | --- | --- | --- | --- | --- |
| Not a  priority | Low  priority | Somewhat  a priority | Neutral | Moderate  priority | High  priority | Essential  priority |
| ☐ | ☐ | ☐ | ☐ | ☐ | ☐ | ☐ |

Why did you choose this rating? (optional)

Additional comments or suggestions (optional)

| Q18 | Privacy-by-design principles embedded in WPV-ONTO's structure, including: data minimisation guidance (annotation of which properties require personal data and which can be anonymised at source); explicit modelling of data subject rights relevant to incident records (access, rectification, erasure); and guidance on lawful bases for processing incident data under GDPR.  Note: WPV-ONTO will likely be used to structure databases containing personal data. Legal and privacy experts on the panel are asked particularly to consider this question. |
| --- | --- |

WPV-ONTO should prioritise:

| 1 | 2 | 3 | 4 | 5 | 6 | 7 |
| --- | --- | --- | --- | --- | --- | --- |
| Not a  priority | Low  priority | Somewhat  a priority | Neutral | Moderate  priority | High  priority | Essential  priority |
| ☐ | ☐ | ☐ | ☐ | ☐ | ☐ | ☐ |

Why did you choose this rating? (optional)

Additional comments or suggestions (optional)

| Q19 | A linkage module enabling WPV-ONTO to connect with adjacent information systems, specifically: electronic health or patient records (linking injury to treatment); justice and police information systems (linking incident to criminal proceedings); human resources systems (linking incident to employment outcomes such as sick leave or role change); and occupational health systems.  Note: Cross-system linkage is a key stated goal of WPV-ONTO and is also the area of highest privacy and legal risk. Consider both the value and the risks when rating this item. |
| --- | --- |

WPV-ONTO should prioritise:

| 1 | 2 | 3 | 4 | 5 | 6 | 7 |
| --- | --- | --- | --- | --- | --- | --- |
| Not a  priority | Low  priority | Somewhat  a priority | Neutral | Moderate  priority | High  priority | Essential  priority |
| ☐ | ☐ | ☐ | ☐ | ☐ | ☐ | ☐ |

Why did you choose this rating? (optional)

Additional comments or suggestions (optional)

| Q20 | Multilingual support built into WPV-ONTO from the outset, including preferred labels and synonyms in at least three EU official languages relevant to the primary user base (e.g., English, French, Dutch/Flemish, German), following the SKOS/OWL annotation property conventions for multilingual ontologies. |
| --- | --- |

WPV-ONTO should prioritise:

| 1 | 2 | 3 | 4 | 5 | 6 | 7 |
| --- | --- | --- | --- | --- | --- | --- |
| Not a  priority | Low  priority | Somewhat  a priority | Neutral | Moderate  priority | High  priority | Essential  priority |
| ☐ | ☐ | ☐ | ☐ | ☐ | ☐ | ☐ |

Why did you choose this rating? (optional)

Additional comments or suggestions (optional)

| Q21 | A formal evaluation framework for WPV-ONTO, specifying: the criteria by which the ontology's coverage, accuracy, and usability will be assessed; the stakeholder groups involved in evaluation (frontline workers, system developers, policymakers, researchers); and a planned review cycle.  Note: Evaluation is often omitted from ontology development protocols but is critical to long-term adoption and scientific credibility. |
| --- | --- |

WPV-ONTO should prioritise:

| 1 | 2 | 3 | 4 | 5 | 6 | 7 |
| --- | --- | --- | --- | --- | --- | --- |
| Not a  priority | Low  priority | Somewhat  a priority | Neutral | Moderate  priority | High  priority | Essential  priority |
| ☐ | ☐ | ☐ | ☐ | ☐ | ☐ | ☐ |

Why did you choose this rating? (optional)

Additional comments or suggestions (optional)

Round 1 — Open Question (for all panellists)

Are there any WPV concepts, definitions, relationships, structural features, or cross-sector considerations that you believe should be subjected to expert panel priority rating in Round 2, but that are not covered by the questions above?

Please describe each suggested item as specifically as possible, including the proposed wording you would like the panel to rate.

Your suggestions for Round 2 questions (optional):

Before you submit

- Please check that you have attempted all 21 questions and completed any open text comments you wished to add.
- Your responses are strictly confidential. Only anonymised aggregate results will be shared with the panel.
- You will receive a summary report of Round 1 results before being invited to complete Round 2.
- Thank you for your expert contribution to WPV-ONTO.
