# Supplementary material for: An Ontology for Workplace Violence: Protocol to Support Cross-Sector Incident Reporting in Public Services
Source: JMIR Res Protoc. 2026 Oct 15;15:e94392. doi: 10.2196/94392 (PMC13720405; doi:10.2196/94392)
Supplement: Supplementary file 4 [file resprot_v15i1e94392_app4.docx]

**Status:** Draft. These search strings will be further refined before the definitive search is run, and the final search strings will be registered on the Open Science Framework (OSF). Each string below is a single continuous string.

1. **PubMed (NLM)**

**Platform:** PubMed (pubmed.ncbi.nlm.nih.gov)

**Field tags:** [tiab] (title/abstract)

**Limits:** Applied through interface filters: publication date 2020-01-01 to date of search; English. Non-peer-reviewed publication types (editorials, letters, comments, news) are excluded at the screening stage.

**Search string:**

(“workplace violence”[tiab] OR “workplace aggression”[tiab] OR “occupational violence”[tiab] OR

“third-party violence”[tiab]) AND (taxonomy[tiab] OR taxonomies[tiab] OR typology[tiab] OR

typologies[tiab] OR classification[tiab] OR classifications[tiab] OR nomenclature[tiab] OR

definition[tiab] OR framework[tiab] OR “incident report”[tiab] OR “incident reports”[tiab] OR

“incident reporting”[tiab] OR coding[tiab] OR instrument[tiab]) AND (healthcare[tiab] OR

“emergency services”[tiab] OR police[tiab] OR “fire service”[tiab] OR “fire services”[tiab] OR

education[tiab] OR “social welfare”[tiab] OR “public transport”[tiab] OR “public transportation”[tiab])

2. **Web of Science, Core Collection**

**Platform:** Web of Science (Clarivate), Advanced Search

**Field tags:** TI= (title), AB= (abstract)

**Limits:** Applied within the string: PY=(2020-2026); LA=(English); DT=(Article OR Review).

**Search string:**

((TI=("workplace violence" OR "workplace aggression" OR "occupational violence" OR

"third-party violence") OR AB=("workplace violence" OR "workplace aggression" OR

"occupational violence" OR "third-party violence")) AND (TI=(taxonomy OR taxonomies OR

typology OR typologies OR classification OR classifications OR nomenclature OR definition

OR framework OR "incident report" OR "incident reports" OR "incident reporting" OR coding

OR instrument) OR AB=(taxonomy OR taxonomies OR typology OR typologies OR classification

OR classifications OR nomenclature OR definition OR framework OR "incident report" OR

"incident reports" OR "incident reporting" OR coding OR instrument)) AND

(TI=(healthcare OR "emergency services" OR police OR "fire service" OR "fire services" OR

education OR "social welfare" OR "public transport" OR "public transportation") OR

AB=(healthcare OR "emergency services" OR police OR "fire service" OR "fire services" OR

education OR "social welfare" OR "public transport" OR "public transportation")))

AND PY=(2020-2026) AND LA=(English) AND DT=(Article OR Review)

3. **ERIC (ProQuest)**

**Platform:** ProQuest, Advanced Search

**Field tags:** TI() (title), AB() (abstract)

**Limits:** Applied through interface: publication date 2020-01-01 to date of search; English; Peer reviewed; Source type: Scholarly Journals.

**Adaptations:** Two substantive adaptations. First, the workplace-violence block is expanded to include education-specific terminology (school violence, violence against teachers, teacher victimization/victimisation, student-to-teacher violence, aggression toward/towards teachers), because the education literature rarely uses the workplace-violence umbrella terms; without this extension, the string returns almost no relevant records. Second, the sector block is omitted because ERIC indexes education literature exclusively. The term 'education' is therefore non-discriminating within it, while excluding relevant records that do not use the word.

**Search string:**

( TI ("workplace violence" OR "workplace aggression" OR "occupational violence" OR "third-party violence"

OR "school violence" OR "violence against teachers" OR "teacher victimization" OR "teacher victimisation" OR

"student-to-teacher violence" OR "aggression toward teachers" OR "aggression towards teachers" ) OR

AB ("workplace violence" OR "workplace aggression" OR "occupational violence" OR "third-party violence" OR

"school violence" OR "violence against teachers" OR "teacher victimization" OR "teacher victimisation" OR

"student-to-teacher violence" OR "aggression toward teachers" OR "aggression towards teachers" ) ) AND

( TI (taxonomy OR taxonomies OR typology OR typologies OR classification OR classifications OR

nomenclature OR definition OR framework OR "incident report" OR "incident reports" OR

"incident reporting" OR coding OR instrument ) OR AB ( taxonomy OR taxonomies OR typology OR typologies OR

classification OR classifications OR nomenclature OR definition OR framework OR "incident report" OR

"incident reports" OR "incident reporting" OR coding OR instrument ) )

4. **APA PsycInfo® (ProQuest)**

**Platform:** ProQuest, Advanced Search

**Field tags:** TI() (title), AB() (abstract)

**Limits:** Applied through interface: publication date 2020-01-01 to date of search; English; Peer reviewed; Source type: Scholarly Journals.

**Search string:**

(TI("workplace violence" OR "workplace aggression" OR "occupational violence" OR

"third-party violence") OR AB("workplace violence" OR "workplace aggression" OR

"occupational violence" OR "third-party violence")) AND (TI(taxonomy OR taxonomies OR

typology OR typologies OR classification OR classifications OR nomenclature OR definition

OR framework OR "incident report" OR "incident reports" OR "incident reporting" OR coding

OR instrument) OR AB(taxonomy OR taxonomies OR typology OR typologies OR classification

OR classifications OR nomenclature OR definition OR framework OR "incident report" OR

"incident reports" OR "incident reporting" OR coding OR instrument)) AND

(TI(healthcare OR "emergency services" OR police OR "fire service" OR "fire services" OR

education OR "social welfare" OR "public transport" OR "public transportation") OR

AB(healthcare OR "emergency services" OR police OR "fire service" OR "fire services" OR

education OR "social welfare" OR "public transport" OR "public transportation"))

5. **Sociological Abstracts (ProQuest)**

**Platform:** ProQuest, Advanced Search

**Field tags:** TI() (title), AB() (abstract)

**Limits:** Applied through interface: publication date 2020-01-01 to date of search; English; Peer reviewed; Source type: Scholarly Journals.

**Adaptations:** The sector block is extended with 'social work' and 'social services', which are the disciplinary terms used in the social-science literature for the sector this protocol refers to as social welfare.

**Search string:**

(TI("workplace violence" OR "workplace aggression" OR "occupational violence" OR

"third-party violence") OR AB("workplace violence" OR "workplace aggression" OR

"occupational violence" OR "third-party violence")) AND (TI(taxonomy OR taxonomies OR

typology OR typologies OR classification OR classifications OR nomenclature OR definition

OR framework OR "incident report" OR "incident reports" OR "incident reporting" OR coding

OR instrument) OR AB(taxonomy OR taxonomies OR typology OR typologies OR classification

OR classifications OR nomenclature OR definition OR framework OR "incident report" OR

"incident reports" OR "incident reporting" OR coding OR instrument)) AND

(TI(healthcare OR "emergency services" OR police OR "fire service" OR "fire services" OR

education OR "social welfare" OR "social work" OR "social services" OR "public transport"

OR "public transportation") OR AB(healthcare OR "emergency services" OR police OR

"fire service" OR "fire services" OR education OR "social welfare" OR "social work" OR

"social services" OR "public transport" OR "public transportation"))
