# Supplementary material for: An Ontology for Workplace Violence: Protocol to Support Cross-Sector Incident Reporting in Public Services
Source: JMIR Res Protoc. 2026 Oct 15;15:e94392. doi: 10.2196/94392 (PMC13720405; doi:10.2196/94392)
Supplement: Supplementary file 5 [file resprot_v15i1e94392_app5.docx]

**Status:** This section documents the second evidence stream described under ‘Targeted review of standards, guidance and grey literature’. This core collection is not exhaustive and additional sources identified during execution will be assessed against the same inclusion criteria and recorded in the source log.

**Table S1.** Core collection of standards, guidance and grey literature

| **Source** | **Type** | **Relevance** | **Access route** |
| --- | --- | --- | --- |
| **Fedris** | National authority | Belgian occupational-injury reporting | https://www.fedris.be |
| **FPS Employment, Labour and Social Dialogue** | National authority | Belgian regulator for psychosocial risks at work, including violence, harassment and third-party aggression | https://employment.belgium.be |
| **ISO 45003:2021** | Standard | Psychosocial risk management | https://www.iso.org/obp/ui/#iso:std:iso:45003:ed-1:v1:en |
| **ILO Guide on Convention No. 190 and Recommendation No. 206** | Standard/instrument | Definitional scope of WPV | https://www.ilo.org |
| **ILO, ICN, WHO, PSI. Framework guidelines** | Guidelines | WPV framework for the health sector | https://www.ilo.org https://www.who.int |
| **EU-OSHA** | Guidelines | OSH risk-management, sector reports | https://osha.europa.eu/en |
| **Eurofound** | Standard/instrument | EU comparative data | https://www.eurofound.europa.eu |
| **WHO** | Guidelines | WPV in the health sector | https://www.who.int |
| **UITP** | Sector federation | Transport framework | https://www.uitp.org |
| **EPSU** | Sector federation | Public-sector framework | https://www.epsu.org |

**Table S2.** Source log for the targeted reviews

| **Source** | **Type** | **Route** | **Terms applied** | **Date accessed** | **Results screened** | **Record included** | **New concepts yielded (Y/N)** |
| --- | --- | --- | --- | --- | --- | --- | --- |
| **Fedris** | National authority | Predefined |  |  |  |  |  |
| **FPS Employment, Labour and Social Dialogue** | National authority | Predefined |  |  |  |  |  |
| **ISO 45003:2021** | Standard | Predefined |  |  |  |  |  |
| **ILO Guide on Convention No. 190 and Recommendation No. 206** | Standard/instrument | Predefined |  |  |  |  |  |
| **ILO, ICN, WHO, PSI. Framework guidelines** | Guidelines | Predefined |  |  |  |  |  |
| **EU-OSHA** | Guidelines | Predefined |  |  |  |  |  |
| **Eurofound** | Standard/instrument | Predefined |  |  |  |  |  |
| **WHO** | Guidelines | Predefined |  |  |  |  |  |
| **UITP** | Sector federation | Predefined |  |  |  |  |  |
| **EPSU** | Sector federation | Predefined |  |  |  |  |  |

*Saturation is assessed after each completed source: extraction stops when two consecutive sources yield no new candidate concepts, definitions, or synonyms relative to those already charted. The source at which saturation was reached will be marked in the "New concepts yielded" column.*
