# Supplementary material for: An Ontology for Workplace Violence: Protocol to Support Cross-Sector Incident Reporting in Public Services
Source: JMIR Res Protoc. 2026 Oct 15;15:e94392. doi: 10.2196/94392 (PMC13720405; doi:10.2196/94392)
Supplement: Supplementary file 6 [file resprot_v15i1e94392_app6.docx]

**Study title:** An Ontology for Workplace Violence: Protocol to Support Cross-Sector Incident Reporting in Public Services

**Principal Investigator:** Iris Steenhout

**Contact:** Iris.Steenhout@vub.be - +32 486386641

**Research Team:** REDM, Vrije Universiteit Brussel, Pleinlaan 2, 1050 Brussels

**Ethics reference:** to be completed

**DPO:** Vrije Universiteit Brussel, Pleinlaan 2 - 1050 Brussels, Belgium. Contact: dpo@vub.be

Dear,

You are invited to take part in a small pilot study testing a new digital form for reporting incidents of workplace violence. The form is based on WPV-ONTO, a shared vocabulary for workplace violence (WPV) in public services, being developed by our research team. We are looking for frontline workers in healthcare and public transport to try the form out and tell us how well it works in practice.

This Participant Information Sheet sets out everything you need to know about the study. Please read it carefully. You are under no obligation to participate, and you may take as much time as you need to decide. If you have any questions, please contact the principal investigator using the details above.

**Section 1: Purpose and Background**

**Why is this study being conducted?**

Incidents of workplace violence are described and coded differently across organizations and sectors, which makes it difficult to compare them, learn from them, or plan prevention. WPV-ONTO is being developed to provide a shared vocabulary that works across healthcare, education, social welfare, policing, emergency response, and public transport. It has been built with the help of an expert panel.

A vocabulary that looks coherent to experts does not necessarily work for the people who have to use it at the end of a shift. The purpose of this pilot is therefore practical: to determine whether real incidents can be recorded using the new form without important details being lost, and whether doing so is quicker or more burdensome than the reporting procedure you currently use.

**What will the results be used for?**

Your feedback will be used directly to revise WPV-ONTO before its first public release. Problems identified in this pilot are logged as formal change requests and reviewed by the project's editorial board; if the form does not meet the standards we have set in advance, it will be revised before release rather than published as it stands.

**Section 2: Who We Are Looking For**

We are recruiting 6 to 8 participants in total, from two sectors: healthcare and public transport. You are eligible to participate if all of the following apply:

- You are currently employed in a frontline public-service role that involves direct contact with members of the public.
- You have been in your current post for at least six months.
- You have previously used your organization's incident reporting system.

You are not eligible if you are a member of the WPV-ONTO expert panel. This is not a reflection on your expertise: the purpose of this pilot is to test the vocabulary with people who did not help build it.

Recruitment takes place through the sector organizations represented on the national committee on aggression against public-service workers. Taking part, or declining to take part, has no bearing on your employment and will not be reported to your employer.

**Section 3: What Does Participation Involve?**

Participation consists of a single session of approximately 60 to 90 minutes, arranged at a time and place that suits you. It may take place at your workplace, at another agreed location, or online. There is only one session; there are no follow-up rounds.

| **Stage** | **Time** | **What you will be asked to do** |
| --- | --- | --- |
| Introduction | ~10 min | Brief explanation of the form and an opportunity to ask questions. No prior training or technical knowledge is required. |
| Encoding task | ~30-45 min | Read eight short written descriptions of workplace violence incidents and record each one using the new form. Where your sector's current reporting form is available, you will record the same descriptions on that form as well, so the two can be compared. |
| Questionnaire | ~5 min | A ten-item standard questionnaire on how easy or difficult the form was to use (the System Usability Scale). |
| Interview | ~15-20 min | A short conversation about which concepts were difficult to apply and whether recording an incident felt quicker or more burdensome than your current procedure. With your permission, this will be audio-recorded so that it can be transcribed accurately. |

**What are the incident descriptions?**

The eight descriptions are written scenarios prepared by the research team. They are based on anonymized accounts of real incidents, rewritten so that no person, organization, or location can be identified, and reviewed by the expert panel to check that they are realistic. They are not accounts of incidents involving you or your colleagues.

You will not be asked to report or describe any incident you have personally experienced. If you wish to draw on your own experience when commenting on the form, that is entirely your choice.

**Section 4: Voluntary Participation and Withdrawal**

Participation is entirely voluntary. You are free to:

- Decline to answer any individual question, or to skip any of the eight descriptions
- Ask for the audio recording to be paused or stopped at any point
- Stop the session at any time, without giving a reason, and without any negative consequence
- Withdraw from the study after the session by contacting the principal investigator

To withdraw, simply email the principal investigator at the address listed at the top of this document. If you withdraw before the final dataset is locked, you may also request deletion of your data. Because the data are anonymized at the point of analysis, full retrospective deletion may not be possible once results have been aggregated; the team will advise you on what is feasible at the time of your withdrawal.

**Section 5: Potential Benefits and Risks**

**Benefits**

There is no direct personal benefit to you from participating. Your contribution will, however, directly shape an open-access resource intended to improve how workplace violence is recorded and prevented across public services. Participants who wish to receive a summary of the pilot findings will receive it once the study is complete.

**Risks**

The risks associated with this study are minimal. You will not be asked about your own experiences of victimization. However, the written scenarios describe incidents of workplace violence, including physical assault, threats, and harassment, and reading them may be sensitizing, particularly if you have experienced something similar. You may skip any scenario, pause at any point, or stop the session entirely, without giving a reason and without penalty.

Should you wish to access occupational mental health support, we encourage you to consult your employer's employee assistance program or an occupational health service. A list of general support resources is provided at the end of this document.

**Section 6: Confidentiality and Anonymity**

- Your responses are recorded under a participant code, not your name
- Contact details are held separately from your responses and will not appear in any dataset used for analysis or publication
- Neither your employer nor your colleagues will be told whether you took part, or what you said
- Any direct quotes included in publications will be reported without attribution and with any identifying detail removed
- Because the pilot involves a small number of participants from two sectors, results will be reported in a way that does not allow individuals to be identified; where a finding could identify a participant, it will be reported at group level only

All data are stored on encrypted, password-protected institutional servers accessible only to named members of the research team. Audio recordings are deleted once transcription has been checked.

**Section 7: How Your Personal Data Will Be Used (GDPR)**

This study is conducted in accordance with Regulation (EU) 2016/679, the General Data Protection Regulation (GDPR).

**What personal data are collected?**

- Professional background (sector, role, years of experience): collected to describe the participant group
- Contact details (name, email address or telephone number): used only to arrange the session and to send you the summary of findings if requested
- Task data: the codes you assign to each scenario, the time taken, and your questionnaire responses
- Interview data: an audio recording, if you consent to it, and the resulting transcript

No special-category personal data are collected. You will not be asked about your health, ethnicity, religion, political opinions, or personal experiences of victimization.

**Legal basis for processing**

**Consent:** the primary basis for processing your contact details, task data, and interview data.

**Public interest / scientific research:** provides a supplementary basis for processing anonymized research data in the public interest, in conjunction with applicable national implementing legislation.

**How long will your data be kept?**

Contact details and individually identifiable responses will be retained for a maximum of five years from the end of the project, in line with good scientific practice guidelines and applicable institutional data retention policy. Anonymized data incorporated into publications will be retained indefinitely as part of the scientific record.

**Will your data be transferred outside the EU/EEA?**

Data are processed within the European Economic Area (EEA).

**Your rights under GDPR**

| **Your right** | **What it means in practice** |
| --- | --- |
| Right of access | Request confirmation that your data are processed and obtain a copy. |
| Right to rectification | Ask us to correct inaccurate or incomplete personal data. |
| Right to erasure | Request deletion of your data, subject to research exemptions under Art. 17(3)(d). |
| Right to restriction | Ask us to limit how your data are used while a dispute is resolved. |
| Right to data portability | Receive your data in a structured, machine-readable format. |
| Right to object | Object to processing based on public interest or legitimate interests. |
| Right to withdraw consent | Withdraw consent at any time without affecting the lawfulness of prior processing. |

You also have the right to lodge a complaint with a data protection supervisory authority. In Belgium, this is:

**Gegevensbeschermingsautoriteit / Autorité de protection des données (GBA/APD)**

Rue de la Presse 35 / Drukpersstraat 35, 1000 Brussels

www.gegevensbeschermingsautoriteit.be | contact@apd-gba.be | +32 2 274 48 00

*You may also contact the supervisory authority in the EU Member State of your habitual residence or place of work.*

**Section 8: Results and Dissemination**

- A summary of the pilot findings will be shared with all participants
- Findings will be submitted for publication in peer-reviewed journals and/or presented at relevant conferences
- Participants will receive copies of any publications resulting from this work
- Changes made to WPV-ONTO as a result of this pilot will be published with the version 1.0 release documentation

No individually identifiable information will appear in any publication.

**Section 9: How to Express Your Interest**

If you are willing to participate, please contact the principal investigator using the details at the top of this document. You will then receive the consent form, and we will arrange a session at a time that suits you.

If you do not wish to participate, no further action is needed. Please simply disregard this invitation.

Thank you sincerely for your time and consideration.

**Complaints and concerns**

If you have any concerns about the conduct of this research that you do not feel comfortable raising with the research team, please contact the Ethics Committee for the Human Sciences of the Vrije Universiteit Brussel: echw@vub.be.

**Support Resources**

While this study does not address personal experiences of victimization, the following organizations offer support related to workplace wellbeing and violence:

| **Organization / resource** | **Contact** |
| --- | --- |
| European Agency for Safety and Health at Work (EU-OSHA) | osha.europa.eu |
| PREVENT (Belgian prevention institute) | www.prevent.be |
| Employee Assistance Program (EAP) | Contact your employer's HR or occupational health service |
| Your occupational health / wellbeing service | Contact your employer |
