# Supplementary material for: An Ontology for Workplace Violence: Protocol to Support Cross-Sector Incident Reporting in Public Services
Source: JMIR Res Protoc. 2026 Oct 15;15:e94392. doi: 10.2196/94392 (PMC13720405; doi:10.2196/94392)
Supplement: Supplementary file 7 [file resprot_v15i1e94392_app7.docx]

**Study title:** An Ontology for Workplace Violence: Protocol to Support Cross-Sector Incident Reporting in Public Services

**Principal Investigator:** Iris Steenhout

**Contact:** Iris.Steenhout@vub.be - +32 486386641

**Research Team:** REDM, Vrije Universiteit Brussel, Pleinlaan 2, 1050 Brussels

**Ethics reference:** to be completed

**DPO:** Vrije Universiteit Brussel, Pleinlaan 2 - 1050 Brussels, Belgium. Contact: dpo@vub.be

*Please read each statement and write your initials in the box beside it if you agree. If you do not agree with a statement, leave the box blank and discuss it with the researcher; you may still be able to take part.*

**Part A: Study Identification**

| **Item** | **Detail** |
| --- | --- |
| Study | WPV-ONTO pilot usability evaluation |
| Data controller | Vrije Universiteit Brussel, Pleinlaan 2, 1050 Brussels, Belgium |
| Principal Investigator | Iris Steenhout (Iris.Steenhout@vub.be) |
| Data Protection Officer | dpo@vub.be |
| Ethics reference | to be completed |
| Information sheet | Appendix 6: Pilot Usability Evaluation - Participant Information Sheet |

**Part B: Declarations**

| **Statement** | **Initials** |
| --- | --- |
| 1. I confirm that I have read and understood the Participant Information Sheet for this study, and that I have had the opportunity to ask questions and have had these answered satisfactorily. |  |
| 2. I understand that my participation is voluntary and that I am free to stop the session or withdraw from the study at any time, without giving a reason and without any negative consequence for me or my employment. |  |
| 3. I confirm that I am currently employed in a frontline public-service role involving direct contact with members of the public, that I have been in post for at least six months, that I have previously used my organization's incident reporting system and that I am not a member of the WPV-ONTO expert panel. |  |
| 4. I agree to take part in a single session of approximately 60 to 90 minutes, in which I will record eight written incident scenarios using a prototype reporting form, complete a short usability questionnaire, and take part in a short interview. |  |
| 5. I understand that the incident scenarios describe workplace violence, that I may find them sensitizing, and that I may skip any scenario, pause, or stop at any point. |  |
| 6. I understand that I will not be asked about my own experiences of victimization. |  |
| 7. I understand that my responses will be recorded under a participant code, that my contact details will be stored separately from my responses, and that neither my employer nor my colleagues will be informed whether I took part or what I said. |  |
| 8. I understand that anonymized quotes from the interview may be used in publications, reported without attribution and with identifying details removed. |  |
| 9. I understand how my personal data will be processed, as described in Section 7 of the Participant Information Sheet, and that I may exercise my rights under the GDPR at any time by contacting the research team or the Data Protection Officer. |  |
| 10. I agree to take part in the study described above. |  |

**Part C: Optional Consent**

*These items are optional. You may take part in the study whether or not you agree to them.*

| **Statement** | **Yes** | **No** |
| --- | --- | --- |
| 11. I agree to the interview being audio-recorded, and understand that the recording will be deleted once the transcript has been checked. |  |  |
| 12. I would like to receive a summary of the pilot findings and copies of any resulting publications. |  |  |

**Part D: Signatures**

|  | **Name (block capitals)** | **Signature** | **Date** |
| --- | --- | --- | --- |
| Participant |  |  |  |
| Researcher taking consent |  |  |  |

*Two copies of this form will be signed. One copy will be given to the participant, together with a copy of the Participant Information Sheet. One copy will be held securely by the research team for the duration of the project and for five years thereafter.*
